# Evolution in key indicators of maternal and child health across the wealth gradient in 41 sub-Saharan African countries, 1986–2019

**DOI:** 10.1186/s12916-023-03183-0

**Published:** 2024-01-08

**Authors:** Yeeun Lee, Sarah Bolongaita, Ryoko Sato, Jesse B. Bump, Stéphane Verguet

**Affiliations:** 1 Department of Global Health and Population, Harvard T.H. Chan School of Public Health, 677 Huntington Avenue, Boston, MA 02115 USA; 2 Bergen Center for Ethics and Priority Setting, Bergen, Norway

**Keywords:** Maternal and child health, Health disparities, Wealth inequality, Sub-Saharan Africa, Equity, Trends

## Abstract

**Background:**

Aggregate trends can be useful for summarizing large amounts of information, but this can obscure important distributional aspects. Some population subgroups can be worse off even as averages climb, for example. Distributional information can identify health inequalities, which is essential to understanding their drivers and possible remedies.

**Methods:**

Using publicly available Demographic and Health Survey (DHS) data from 41 sub-Saharan African countries from 1986 to 2019, we analyzed changes in coverage for eight key maternal and child health indicators: first dose of measles vaccine (MCV1); Diphtheria-Pertussis-Tetanus (DPT) first dose (DPT1); DPT third dose (DPT3); care-seeking for diarrhea, acute respiratory infections (ARI), or fever; skilled birth attendance (SBA); and having four antenatal care (ANC) visits. To evaluate whether coverage diverged or converged over time across the wealth gradient, we computed several dispersion metrics including the coefficient of variation across wealth quintiles. Slopes and 5-year moving averages were computed to identify overall long-term trends.

**Results:**

Average coverage increased for all quintiles and indicators, although the range and the speed at which they increased varied widely. There were small changes in the wealth-related gap for SBA, ANC, and fever. The wealth-related gap of vaccination-related indicators (DPT1, DPT3, MCV1) decreased over time. Compared to 2017, the wealth-gap between richest and poorest quintiles in 1995 was 7 percentage points larger for ANC and 17 percentage points larger for measles vaccination.

**Conclusions:**

Maternal and child health indicators show progress, but the distributional effects show differential evolutions in inequalities. Several reasons may explain why countries had smaller wealth-related gap trends in vaccination-related indicators compared to others. In addition to service delivery differences, we hypothesize that the allocation of development assistance for health, the prioritization of vaccine-preventable diseases on the global agenda, and indirect effects of structural adjustment programs on health system-related indicators might have played a role.

**Supplementary Information:**

The online version contains supplementary material available at 10.1186/s12916-023-03183-0.

## Background

Discussing global health outcomes in terms of aggregate trends can be useful for communicating with donors or the public, but this might obscure important distributional information. Understanding distributional effects and whether the underlying distributions of health outcomes thinned or widened over time is crucial to identify health inequalities, their drivers, and ways to improve health equity. By aggregating health outcomes, one might be prone to misunderstanding the situation of certain groups such as the poorest, which obscures opportunities to target interventions towards those who need them most. While there are many reasons why disaggregating data to better understand health inequalities is important, perhaps the most compelling reason is that some health inequalities may be unfair and avoidable. They can stem from historical factors and may be exacerbated by policies and systems that support the unjust distribution of resources and opportunities. This is why reducing inequalities is central to the United Nations Sustainable Development Goals (SDGs) [1]. SDG 10 calls for reducing inequality both within and between countries, emphasizing several dimensions of inequality, such as by income, age, gender, race, and ethnicity, that can deepen inequalities in health [2, 3].

Did infant mortality decrease more quickly among the rich than the poor? Was there an equitable uptake of childhood immunizations across socioeconomic groups? Important questions such as these are overlooked when analysts fixate on aggregate trends. As an illustration, a 2020 newsletter about child survival began with how “the global under-5 mortality rate has dropped by 59%” since 1990 [4]. The newsletter reported differences in child survival by region, but missed distributional effects by residence, income, or education. Perhaps child survival improved in the richest groups and worsened in the poorest groups, but the average may falsely imply that it improved for everyone.

It remains unclear if aggregate trends have corresponded to disparities that have increased or decreased across wealth quintiles, either between or within countries. For example, the under-five mortality rate in sub-Saharan Africa (SSA) from 1990 to 2020 shows a steep declining trend, meaning that under-five mortality improved significantly during this period [5]. However, it remains unclear for whom in particular under-five mortality reduced the most and if any group was left behind. Differing healthcare utilization rates across wealth quintiles further could have impacted whether health outcomes diverged or converged over time across the wealth gradient. For instance, full immunization coverage or the percentage of children aged 12–23 months who received all 8 basic vaccinations (single dose of Bacille Calmette-Guérin (BCG); three doses of diphtheria, pertussis, and tetanus (DPT); three doses of oral polio vaccine; and one dose of measles vaccine) greatly differs between the top and bottom wealth quintiles in several sub-Saharan African countries [6].

As example, the poorest children in the Democratic Republic of the Congo (DRC) are much less likely to receive all basic vaccinations compared to the richest children. In 2014, 36% of children in the bottom wealth quintile received all 8 basic vaccinations compared to 65% of children in the top wealth quintile. This is particularly damaging as poor children also tend to have greater exposure to risk factors and a greater vulnerability to worse health outcomes (e.g., wasting and stunting are more prevalent in this group) [5]. Similar differences were seen in Ethiopia and Nigeria. In Ethiopia (2019 data), the rate of the top quintile was more than double that of the bottom quintile (67 vs. 26%) [5]. In Nigeria (2018 data), the rate of the top quintile was around four times the rate of the bottom quintile (59 vs. 15%) [5]. Utilization rates in skilled birth attendance also vary widely by quintile: in the DRC, 66% of those in the bottom quintile compared to 98% of those in the top quintile; similarly, in Ethiopia, with 22 vs. 87%; and Nigeria, with 12 vs. 87% [5].

Studies analyzing health inequality in low- and middle-income countries (LMICs) have focused on under-five mortality, with mixed findings [7–9]. Alongside mortality, understanding patterns of convergence or divergence across the wealth gradient for various health indicators in a systematic manner remains important. Although mortality is an important and commonly used indicator, examining a wide range of health outcomes allows us to evaluate multiple health system components that can contribute to the health status of individuals and identify key determinants of health. This is what we propose to do in this paper, while focusing on selected maternal and child health (MCH) indicators. We examine trends in eight MCH indicators across the wealth gradient in a large sample of 41 sub-Saharan African countries which have some of the highest rates of maternal mortality and under-five mortality, based on aggregate trends [10, 11]. In 2017, the maternal mortality ratio (MMR) in SSA was 534 per 100,000 live births compared to the world average (including SSA) of 211 per 100,000 [12], and in 2019, under-five mortality in SSA was 76 per 1000 live births compared to the world average of 38 per 1000. SSA also presents lower rates of maternal care and children receiving immunizations compared to the global average [13, 14]. Although the Demographic and Health Survey (DHS) provides disaggregate level data by wealth quintile, it remains unclear how various MCH outcomes have improved and by how much among the rich compared to the poor in SSA. To understand the wealth gradient trends of key MCH indicators, we used DHS data to calculate dispersion metrics and visualized longitudinal trends across wealth quintiles and countries. This allowed us to see whether convergence had occurred and to understand whether health indicators were becoming more equitable or not.

## Methods

### Data sources

Publicly available DHS data from 41 sub-Saharan African countries were obtained for the years 1986 to 2019 inclusive (although not all years for all countries were available) through DHS STATCompiler, which provides aggregate statistics from DHS standard surveys (nationally representative household surveys). These datasets provided coverage and utilization estimates by wealth quintile (as well as aggregate coverage). In any given country, DHS wealth indices are developed by measuring constructs that represent economic status (e.g., furniture, land, housing, flooring type, type of toilet facility) and using principal components analysis to assign a weight to each element. Quintiles are then constructed based on a country’s population distribution whereby individuals are assigned a household wealth index score, arranged by their score, and the distribution is cut at five points that form 20% divisions [15, 16]. The analysis includes information from 1,587,022 households from across 188 DHS and 940 wealth quintile observations.

### Indicator selection

To select indicators, we began with Hogan and colleagues (2018) who proposed an index of 16 tracer indicators from various categories (reproductive, maternal, newborn, and child health; infectious diseases; non-communicable diseases (NCDs); and service capacity and access) to measure progress toward SDG 3, the health SDG which includes a focus on universal health coverage [17]. The MCH indicators reviewed in our analysis did not include all of the tracer indicators proposed by Hogan and colleagues. Indeed, we did not intend to be exhaustive but rather to demonstrate trends illustrative of MCH services. For example, care-seeking for children with suspected pneumonia is a tracer indicator proposed by Hogan and colleagues, yet it was not available in the DHS STATCompiler. Pneumonia is a form of acute respiratory infection and therefore we looked at care-seeking for acute respiratory infection instead. Drawing from this work [17], we selected eight indicators of health services coverage (listed in Table 1) [6]. These eight indicators were selected due to their importance in tracking global progress in the MCH field and the completeness of the publicly available data.

**Table 1:** Indicator names and definitions

Acronym	Indicator	Definition
ANC	Antenatal care	The percentage of women who had four antenatal care visits prior to delivery
SBA	Skilled birth attendance	The percentage of live births assisted by a skilled provider which includes a doctor, nurse, midwife, and auxiliary nurse or midwife
Measles	1st dose of measles vaccine	The percentage of children aged 12–23 months who had received measles vaccine
DPT1	1st dose of Diphtheria-Pertussis-Tetanus vaccination	The percentage of children aged 12–23 months who had received the 1st dose of DPT
DPT3	3rd dose of Diphtheria-Pertussis-Tetanus vaccination	The percentage of children aged 12–23 months who had received the 3rd dose of DPT
Fever	Fever care	The percentage of children with fever for whom advice or treatment was sought from a health facility or provider
Diarrhea	Diarrhea care	The percentage of children with diarrhea who were taken for treatment to a health facility
ARI	Acute respiratory infection care	The percentage of children with ARI for whom advice or treatment was sought from a health facility or health provider

### Analysis

For each indicator (listed in Table 1), we evaluated whether coverage converged over time across the wealth gradient and by country.

To measure these changes in inequality, we chose three dispersion metrics that we calculated across all eight indicators: the absolute difference between the top and bottom wealth quintiles, the relative difference between the top and bottom wealth quintiles or the difference in coverage between the top and bottom quintiles divided by the top quintile, and the coefficient of variation (CoV) across all quintiles (our dispersion metric). CoV is the standard deviation divided by the mean. Five-year moving averages were calculated and plotted to identify overall long-term trends while accounting for potential individual country-level fluctuations.

Additionally, a linear model was run to extract slope coefficients to measure the rate of change in coverage over time (not to infer the effect of wealth or of any other covariate on coverage; see the “Discussion” section). We examined linearity using scatter plots. A linear model regressing year on coverage was conducted and summary statistics including slope coefficients and standard errors were extracted.

All analyses were conducted using RStudio (version 1.4).

## Results

**Fig. 1:**
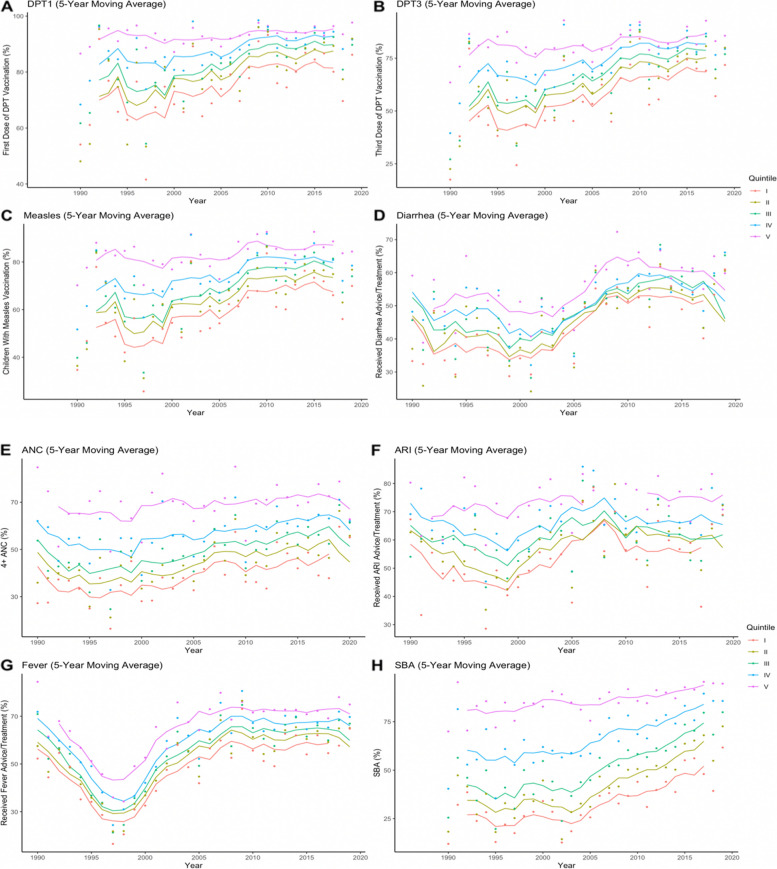
Five-year moving average (1986 to 2019) for eight indicators in sub-Saharan Africa. *DPT1*, first-dose Diphtheria, Tetanus, Pertussis; *DPT3*, third-dose Diphtheria, Tetanus, Pertussis; *ANC*, antenatal care; *ARI*, acute respiratory infection; *SBA*, skilled birth attendance

**Fig. 2:**
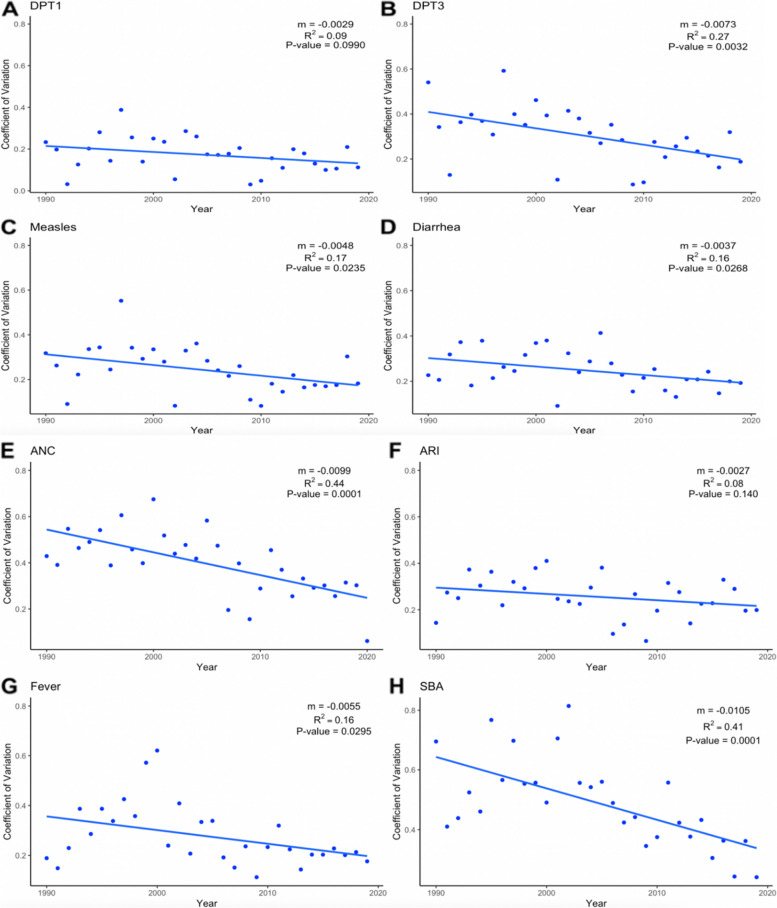
Coefficient of variation values and trends for eight indicators in sub-Saharan Africa, 1986–2019. *DPT1*, first-dose Diphtheria, Tetanus, Pertussis; *DPT3*, third-dose Diphtheria, Tetanus, Pertussis; *ANC*, antenatal care; *ARI*, acute respiratory infection; *SBA*, skilled birth attendance. Note: m (slope) is estimated per year

**Fig. 3:**
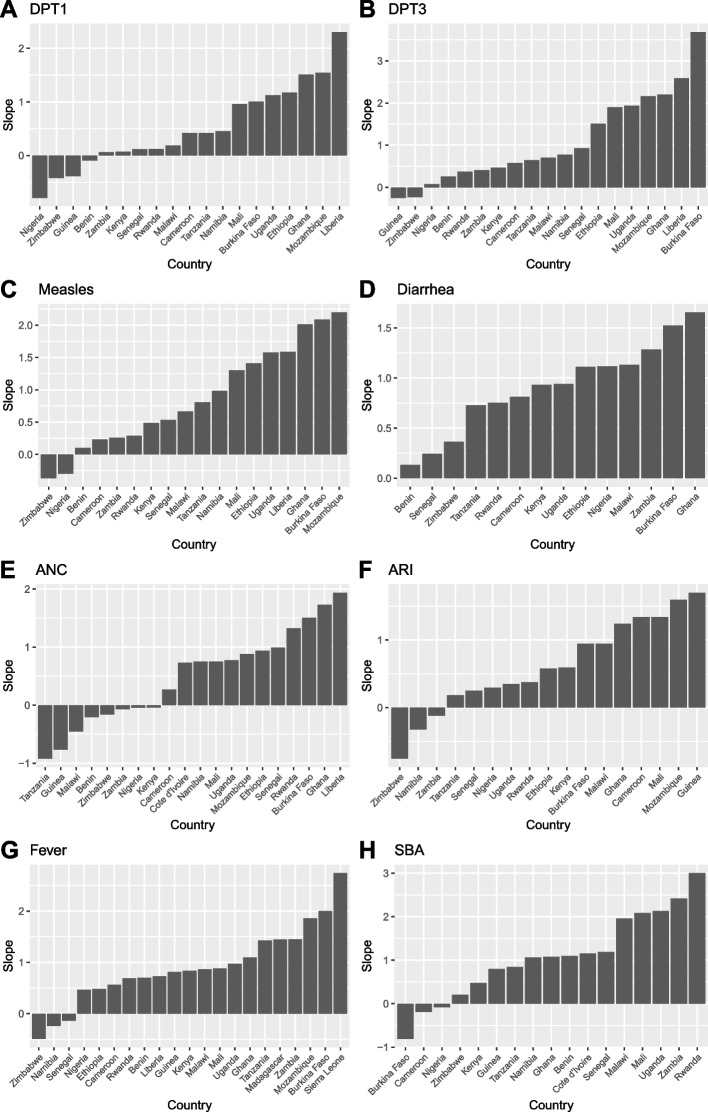
Rate of change in coverage of the bottom quintile for eight indicators in sub-Saharan Africa from 1986 to 2019. Includes countries with more than four surveys. Slope: Average percentage point change between 1986 and 2019 for those in the bottom quintile from 1986 to 2019. *DPT1*, first-dose Diphtheria, Tetanus, Pertussis; *DPT3*, third-dose Diphtheria, Tetanus, Pertussis; *ANC*, antenatal care; *ARI*, acute respiratory infection; *SBA*, skilled birth attendance

Four indicators (ANC, fever, diarrhea, and ARI) had data from 39 countries and four indicators (SBA, measles, DPT1, DPT3) had data from 41 countries. The survey years ranged from 1986 to 2019. Thirty-four countries had 2 or 3 surveys and 21 countries had 4 or more surveys (Table 2). Both sample sizes (at least 2 or 3 surveys and 4 or more surveys) were used for the scatter and trend line plots (Additional file 1: Figure A9), the 5-year moving average plots (Fig. 1), the minimum and maximum plots (Additional file 1: Figure A10), the CoV plots (Fig. 2), the individual country regression plots (Additional file 1: Figure A1), the absolute and relative difference plots (Additional file 1: Figures A3 and A4), and the combined country linear regression plots (Additional file 1: Figure A5). An analysis examining the rate of change in coverage within the bottom quintile was done separately for countries with at least 2 surveys (Additional file 1: Figure A2) and countries with at least 4 surveys (Fig. 3). The density plots of rates of change in coverage within the bottom quintile were conducted for countries with at least 4 data points (Additional file 1: Figure A6). There were around 150 country-year surveys for each indicator, although some had more—there were 170 country-years for fever—and some had less such as ARI, with 133 country-years. Average coverage ranged from 51 to 84% across all indicators. Service coverage within indicators varied widely, for instance, from 1 to 100% for SBA and 3 to 100% for DPT3 (Table 3).

**Table 2:** Indicators presented with the years and number of countries available

Indicator	Years	Countries	Countries with 2 + surveys	Countries with 4 + surveys
ANC	1990–2019	39	34	22
SBA	1986–2019	41	35	20
Measles	1986–2019	41	34	21
DPT1	1986–2019	41	34	21
DPT3	1986–2019	41	34	21
Fever	1990–2019	39	34	22
Diarrhea	1990–2019	39	34	20
ARI	1990–2019	39	32	19

*ANC* antenatal care, *SBA* skilled birth attendance, *DPT1* first-dose Diphtheria, Tetanus, Pertussis, *DPT3* third-dose Diphtheria, Tetanus, Pertussis, *ARI* acute respiratory infection

**Table 3:** Number of country-years and descriptive measures of coverage

Indicator	Country-years	Mean (%)	Median (%)	Range (minimum, maximum) (%)	Range (quartile 1, quartile 3) (%)	SD (%)
ANC	153	51.8	53.7	(1.5, 98.9)	(37.3, 67.8)	21.0
SBA	152	57.3	58.2	(1.0, 100.0)	(35.4, 81.4)	26.3
Measles	151	70.8	75.7	(8.2, 99.3)	(59.6, 85.6)	18.9
DPT1	151	84.0	90.9	(9.9, 100.0)	(76.7, 95.8)	16.6
DPT3	151	68.9	74.7	(3.3, 100.0)	(55.4, 86.7)	22.2
Fever	170	58.8	61.7	(3.5, 95.2)	(47.5, 72.4)	18.2
Diarrhea	139	50.7	50.7	(15.5, 83.2)	(39.6, 62.8)	14.9
ARI	133	62.1	64.7	(11.5, 97.0)	(50.0, 75.0)	17.3

*ANC* antenatal care, *SBA* skilled birth attendance, *DPT1* first-dose Diphtheria, Tetanus, Pertussis, *DPT3* third-dose Diphtheria, Tetanus, Pertussis, *ARI* acute respiratory infection, *SD* standard deviation

There were differences in coverage between quintiles across the eight indicators and varying timepoints. As expected, the top quintile was better off than the bottom quintile. On average, for all indicators and years, the top quintile had the highest coverage, and the bottom quintile the lowest (Additional file 1: Figure A9). Average coverage increased for all quintiles, indicators, and years, although the range of coverage values and the speed at which they increased varied widely (Fig. 1). Although there were minimal changes in the wealth-related gap (difference between top and bottom quintile) of ANC, SBA, and fever over time, the wealth-related gap of SBA and ANC was larger than the wealth-related gap of fever (Fig. 1). For vaccine-related indicators (DPT1, DPT3, MCV1), the wealth-related gap was larger in earlier years and decreased as time progressed. For example, in 1995, the difference in coverage (using 5-year moving averages) between top and bottom quintiles for ANC was 35 percentage points and in 2017 it was 28 percentage points. Comparably, in 1995, the difference in coverage between top and bottom quintiles for measles vaccination was 36 percentage points and in 2017 19 percentage points. In 22 years, the wealth-related gap in coverage between the richest and poorest quintiles decreased by 7 percentage points for ANC and 17 percentage points for measles vaccination. Similarly, the wealth gap between the richest and poorest quintiles in 1992 was 9 percentage points larger for SBA and 15 percentage points larger for DPT vaccination compared to 2016.

In general, the CoV indicated that inequalities in coverage of all indicators had decreased over time, albeit some decreased more quickly than others (Fig. 2). This downward trend was still observed when examining CoV by quintile (Additional file 1: Figure A7), indicating progress across wealth groups in maternal and child health over time in SSA. The decrease varied by quintile with the greatest decrease seen among those in the bottom quintile. For example, inequalities in ANC and SBA (slopes of − 0.0099 and − 0.0110, respectively) decreased more rapidly than inequalities in all other indicators whereas inequalities in ARI and DPT1 (slopes of − 0.0027 and − 0.0029) decreased more slowly compared to all other indicators. Despite more rapid wealth-related gap reductions in ANC and SBA in relative terms, the absolute gap between the top and bottom quintiles in 2019 was larger among these indicators than for vaccination-related indicators.

Changes in coverage over time also widely varied by country. For most countries, the rate of change in coverage for the bottom quintile varied widely by indicator. A given country may have had outcomes either favorable to the poor or the rich depending on the indicator. Figure 3 shows that several countries had increases in coverage within the bottom quintile, implying a smaller wealth-related gap and progress in key MCH indicators over time. When analyzing all countries with two or more DHS surveys, as an illustration, the bottom quintile in Eritrea had improving health outcomes across four indicators: DPT1, DPT3, MCV1, and diarrhea care (Additional file 1: Figure A2). Similarly, Sierra Leone’s bottom quintile also had improving outcomes across four indicators: ANC, ARI, fever care, and SBA. Both countries had the largest positive slope for the bottom quintile across those indicators. Conversely, certain countries had inequality increases across several indicators. As an example, Zimbabwe’s bottom quintile had worsening health outcomes across various indicators. For DPT1, DPT3, MCV1, ANC, and fever care, Zimbabwe had large negative slopes for the bottom quintile (Fig. 3).

When looking at the absolute difference in coverage between top and bottom quintiles over time, some indicators had decreasing steep slopes including DPT1, DPT3, MCV1, diarrhea, and SBA. That is, coverage between bottom and top quintiles converged rapidly, resulting in smaller gaps. However, other indicators such as antenatal care, ARI, and fever stayed flat (Fig. A3). Similar trends were seen when looking at relative differences between the top and bottom quintiles over time (Additional file 1: Figure A4). When examining linear trends for all countries combined, the distances between the trend lines varied by indicator, with the largest distances seen in the SBA trend lines (Additional file 1: Figure A5). Lastly, the density plots of each indicator showed that among countries with at least 4 data points, the slopes of the bottom quintile ranged from greater than 0 to 2% per year for most countries (Additional file 1: Figure A6).

## Discussion

This paper has assessed how improvements in eight MCH indicators were distributed over time in SSA, by wealth quintile, during the period 1986–2019. Improvements were seen across all wealth quintiles and indicators, although some indicators experienced more rapid improvements than others. While wealth-related gaps were reduced across all indicators, several patterns were seen in terms of changes in wealth-related gaps: some gaps remained consistently large, some consistently small, and some began large yet became smaller over time. We focused our analysis on the bottom and top quintiles for simplicity and because there is often great interest in examining the poorest 20% and richest 20%—as such the analysis provides a simple intuitive manner to track health-related inequality in countries. Also, the evolutions among the bottom and top quintiles can somewhat capture the evolutions in adjacent quintiles.

Some countries had decreases in inequality across several indicators while others had increases. For example, in Eritrea, there were inequality decreases across many indicators. This is perhaps related to specific programs. For instance, it was estimated that around 70% of the population had access to primary care services within 10 km by 1999 [18]; community members and teachers were trained to be health workers and in 2020, around 85% of health facilities provided routine immunization services 6 days a week; furthermore, mobile clinics are used in rural areas and routine immunization services are conducted for nomadic groups [19, 20].

Similarly, Jalloh et al. found that Sierra Leone’s 2010 Free Healthcare Initiative (FHCI) improved access to maternal health services, which decreased inequalities in ANC and postnatal care [21]. The 2010 FHCI eliminated user fees for pregnant women, lactating women, and children. However, Jalloh et al. also found that FHCI did not reduce inequalities in SBA [21]. Witter et al. found that FHCI improved ARI outcomes related to geographic inequities [22]. Conversely, in Zimbabwe, there was an increase in inequalities across several indicators which were likely impacted by the country’s economic crises since 1990, which resulted in the deterioration of healthcare infrastructure limiting access to services especially for the poorest rural households [23]. In 1991, Zimbabwe implemented the Economic Structural Adjustment Program [24, 25], which led to temporary increases in user fees [26]. Additionally, the economic crisis led to decreases in development assistance to the country in the early 2000s, potentially affecting immunization efforts and health financing, especially for lower-income groups [27]. From 1999 to 2002, donor financing of national health expenditures decreased from 13 to 1% [23].

Some findings in this analysis are corroborated in the literature. For instance, coverage gaps across quintiles are smaller among vaccination-related indicators compared to health-system dependent indicators (e.g., SBA, ANC). One study analyzed 12 MCH interventions over 2000–2008 using national surveys of 54 countries and found that SBA and 4 + ANC visits were among the most unequal indicators. DPT and MCV1 were hypothesized to be more equitable because vaccines are often free whereas health facility services require out-of-pocket spending [28]. A similar study analyzed data from 1990 to 2006 using 54 countries and found large wealth-related gaps for SBA and small gaps in immunizations [29]; and another study analyzing 28 countries from 2000 to 2008 had similar findings [30]. ANC and SBA had larger gaps between the top and bottom quintiles than for vaccination-related indicators. However, the trends in inequality across these indicators varied widely by country (Additional file 1: Figure A1). Tsegaye et al. evaluated ANC in Ethiopia from 2000 to 2019 and found that overall coverage increased over time with decreasing inequalities [31]. By contrast, inequality in ANC services increased from 2003 to 2014 in Ghana [32].

A possible explanation might be that structural adjustment programs in SSA could be associated with larger wealth-related gaps for indicators more dependent on the health system. Several studies have found that such programs negatively impacted healthcare access: one study pointed that SBA in Uganda decreased from 52 to 38% after the implementation of a structural adjustment loan [33]; another estimated that in Kenya, outpatient visits decreased by 52% in 1989 after a mandate led to a $0.33-equivalent fee per visit [34]; and in Tanzania, user fees implemented in 1994 led to a 53% decrease in public hospital visits [34]. Throughout SSA, structural adjustment programs created conditions that reduced wages for public employees and placed restrictions on the size of the public workforce. In turn, this led many healthcare workers to leave their countries, resulting in a decrease in quality health professionals in SSA [33, 35].

One explanation for the prominent coverage drop in fever care from 1995 to 2000 could be due to the redirecting of health funding from other diseases such as malaria toward HIV/AIDS. Lordan et al. found that from 1991 to 2007, malaria funds were displaced whereas tuberculosis (TB) funds were not, likely due to the strong connection between TB and HIV [36]. Decreased funding for malaria could have impacted access to fever care services (Fig. 1G).

When looking at indicators, those related to immunization (DPT1, DPT3, and MCV1) had patterns indicating smaller wealth-related gaps compared to those (a priori) more dependent on the health system (SBA, ANC, ARI, fever, and diarrhea). This could be due to the prioritization of vaccine-preventable diseases in the global development agenda evidenced by different programs such as WHO’s Expanded Programme on Immunization (EPI) and the 1984 Universal Childhood Immunization initiative jointly led by WHO and UNICEF. Established in 1974 as part of the smallpox eradication efforts, EPI aimed to develop global immunization programs and initially focused on DPT, poliomyelitis, measles, and TB [37]. Worldwide vaccination rates increased after the introduction of both programs, and from 1980 to 1990, DPT3 coverage in the poorest countries increased from 5 to 62% [38]. Similarly, Gavi, the Vaccine Alliance, was established in 2000 to increase the accessibility of vaccines in LMICs. Using a quasi-experimental approach, Jaupart et al. estimated that from 2000 to 2016, Gavi’s programs allowed for significant vaccination coverage increases among eligible LMICs, particularly in SSA [39]. Although there are four vaccines for rotavirus diarrhea, there are several system-level and environmental factors that contribute to diarrhea not caused by rotavirus [40, 41]. Ikilezi et al. also found weak evidence between increased government spending and decreased incidence of diarrhea in LMICs, further demonstrating the complexities and multifaceted challenges of diarrheal disease management [42].

Another reason for the variation between vaccination versus non-vaccination indicators could be due to differences in the amount of development assistance for health (DAH). In 1990, $300 million was allocated towards vaccines compared to $44 million for health system support in the areas of infectious diseases and maternal, newborn, and child health; in 2000, $400 million for vaccines compared to $238 million for health system support; and then in 2010, $1.6 billion compared to $861 million [43]. From 2000 to 2017, SSA received the largest portion of DAH funds out of all world regions; and in 2017, total immunization spending in SSA was similarly comprised of government spending and DAH (45% and 48%, respectively), whereas in all other regions (except South Asia), government spending accounted for most of the spending [42]. Future immunization efforts in SSA will continue to be heavily dependent on donor funding, which may prevent completing the unfinished agenda of immunization and creating resilient health systems in SSA more broadly [44, 45].

Nevertheless, there are some limitations to this analysis. First, while using DHS data allows for the consistent inclusion of many countries, these surveys are subject to recall bias. Second, since DHS are conducted around every 5 years in each country, annual estimates for the same country were not available, and having data for the same country-years was also not possible [46]. Third, averages were used to plot overall trends within SSA, which can be impacted by a few countries with either very high or very low coverage. Fourth, the analysis examined eight key MCH indicators, while numerous additional health services coverage and health system indicators could be studied while applying the same approach. Fifth, some findings might be explained by other factors such as maternal education. However, here, we were interested in analyzing trends solely by wealth. We could also not infer the effect of wealth on coverage of different MCH indicators since we merely use linear regressions to measure the rate of change in coverage over time. Lastly, coverage in earlier surveys could have been impacted by potential data quality issues, and therefore, changes comparing coverage in earlier (1986–2003) and later (2015–2019) periods could partly reflect improved data quality [47, 48]. For example, women in lower quintiles are more likely to have less education and live separately from their children due to child fostering to wealthier relatives. It was found that compared to mothers with higher education, mothers with lower education were more likely to underreport diarrhea that had terminated 2–14 days before the interview in DHS I (1984–1989) [48]. Similarly, Pullum (2008) found that mothers living separately from their children were more likely to report “no” to symptoms of diarrhea in DHS III and Measure DHS (1993–2003) perhaps because that is a preferable response [47]. If underreporting symptoms is related to underreporting care-seeking, the estimates for diarrhea among lower quintiles may be higher than the estimates we used in the earlier period (1986–2003) and the wealth gap may be smaller than what we estimated. Therefore, the improvements we report over time may be smaller since the difference between the wealth gap in more recent years (2015–2019) compared to earlier years (1986–2003) may be smaller.

## Conclusions

The main objective of this research was to understand how MCH improvements were distributed across the wealth gradient in SSA and highlight the limitations of aggregate trends. As expected, generalized improvements show progress, but the distributional effects show differential decreasing inequalities across indicators. In 2019, the coverage gap between top and bottom quintiles was smaller among vaccination-related indicators, for which we proposed several reasons including DAH allocation, structural adjustment programs, and prioritization of immunization on the global health agenda.

### Supplementary Information

**Additional file 1.** Supplementary tables that summarize key indicator information and figures that show trends over time. For example, tables that include information of the countries examined by indicators and years of data available and summary measures of coverage by indicators. Among many other figures, it includes indicator trend figures for six of the most populous countries in sub-Saharan Africa and plots with the rate of change in coverage for those in the lowest quintile by indicator.

## Data Availability

The data are available in the DHS STATCompiler website: https://www.statcompiler.com/en/.

## Abbreviations

ANC: Antenatal care
ARI: Acute respiratory infection
BCG: Bacille Calmette-Guérin
CoV: Coefficient of variation
DAH: Development assistance for health
DHS: Demographic and Health Survey
DPT1: Diphtheria-Pertussis-Tetanus (DPT) first dose
DPT3: Diphtheria-Pertussis-Tetanus (DPT) third dose
DRC: Democratic Republic of the Congo
EPI: Expanded Programme on Immunization
FHCI: Free Healthcare Initiative
LMIC: Low-middle-income countries
MCH: Maternal and child health
MCV1: Measles vaccine first dose
MMR: Maternal mortality ratio
NCD: Non-communicable diseases
UNICEF: United Nations Children’s Fund
SBA: Skilled birth attendance
SDG: Sustainable Development Goals
SSA: Sub-Saharan Africa
WHO: World Health Organization
