# Supplementary material for: Evolution in key indicators of maternal and child health across the wealth gradient in 41 sub-Saharan African countries, 1986–2019
Source: BMC Med. 2024 Jan 8;22:21. doi: 10.1186/s12916-023-03183-0 (PMC10775589; doi:10.1186/s12916-023-03183-0)
Supplement: Supplementary file 1 — Additional file 1. Supplementary tables that summarize key indicator information and figures that show trends over time. For example, tables that include information of the countries examined by indicators and years of data available and summary measures of coverage by indicators. Among many other figures, it includes indicator trend figures for six of the most populous countries in sub-Saharan Africa and plots with the rate of change in coverage for those in the lowest quintile by indicator. [file 12916_2023_3183_MOESM1_ESM.pdf]

Additional File

Evolution in key indicators of maternal and child health across the wealth gradient in 41 sub-Saharan African countries, 1986–2019

Yeeun Lee, Sarah Bolongaita, Ryoko Sato, Jesse Bump, Stéphane Verguet

Table A1. Countries presented with the indicators and years available.

Country	Indicators	Year(s)
Angola		
	ANC	2015
	SBA	2006, 2015
	Measles	2015
	DPT1	2015
	DPT3	2015
	Fever	2006, 2011, 2015
	Diarrhea	2015
	ARI	–
Benin		
	ANC	2001, 2006, 2012, 2017
	SBA	2001, 2006, 2012, 2017
	Measles	1996, 2001, 2006, 2012, 2017
	DPT1	1996, 2001, 2006, 2012, 2017
	DPT3	1996, 2001, 2006, 2012, 2017
	Fever	2001, 2006, 2012, 2017
	Diarrhea	2001, 2006, 2012, 2017
	ARI	2001
Botswana		
	ANC	–
	SBA	1988
	Measles	1988
	DPT1	1988
	DPT3	1988
	Fever	–
	Diarrhea	–
	ARI	–
Burkina Faso		
	ANC	1993, 1999, 2003, 2010, 2014
	SBA	1993, 1999, 2003, 2010
	Measles	1993, 1999, 2003, 2010

	DPT1	1993, 1999, 2003, 2010
	DPT3	1993, 1999, 2003, 2010
	Fever	1993, 1999, 2003, 2010, 2014, 2017
	Diarrhea	1993, 1999, 2003, 2010
	ARI	1993, 1999, 2003, 2010
Burundi		
	ANC	2010, 2016
	SBA	2010, 2016
	Measles	2010, 2016
	DPT1	2010, 2016
	DPT3	2010, 2016
	Fever	2010, 2012, 2016
	Diarrhea	2010, 2016
	ARI	2010, 2016
Cameroon		
	ANC	1991, 2004, 2011, 2018
	SBA	1991, 2004, 2011, 2018
	Measles	1991, 1998, 2004, 2011, 2018
	DPT1	1991, 1998, 2004, 2011, 2018
	DPT3	1991, 1998, 2004, 2011, 2018
	Fever	1991, 2004, 2011, 2018
	Diarrhea	1991, 2004, 2011, 2018
	ARI	2004, 2011
Central African Republic		
	ANC	–
	SBA	–
	Measles	1994
	DPT1	1994
	DPT3	1994
	Fever	–
	Diarrhea	–
	ARI	–
Chad		
	ANC	1997, 2004, 2014
	SBA	1997, 2004, 2014
	Measles	1997, 2004, 2014
	DPT1	1997, 2004, 2014
	DPT3	1997, 2004, 2014
	Fever	1997, 2004, 2014
	Diarrhea	1997, 2004, 2014

	ARI	1997, 2004, 2014
Comoros		
	ANC	2012
	SBA	2012
	Measles	1996, 2012
	DPT1	1996, 2012
	DPT3	1996, 2012
	Fever	2012
	Diarrhea	2012
	ARI	–
Congo		
	ANC	2005, 2011
	SBA	2005, 2011
	Measles	2005, 2011
	DPT1	2005, 2011
	DPT3	2005, 2011
	Fever	2005, 2011
	Diarrhea	2005, 2011
	ARI	2011
Democratic Republic of the Congo		
	ANC	2007, 2013
	SBA	2007, 2013
	Measles	2007, 2013
	DPT1	2007, 2013
	DPT3	2007, 2013
	Fever	2007, 2013
	Diarrhea	2007, 2013
	ARI	–
Cote d'Ivoire		
	ANC	1998, 2005, 2012
	SBA	1998, 2005, 2012
	Measles	1998, 2012
	DPT1	1994, 1998, 2012
	DPT3	1994, 1998, 2012
	Fever	1998, 2012
	Diarrhea	1998, 2012
	ARI	2012
Eritrea		
	ANC	2002
	SBA	2002

	Measles	1995, 2002
	DPT1	1995, 2002
	DPT3	1995, 2002
	Fever	2002
	Diarrhea	2002
	ARI	2002
Eswatini		
	ANC	2006
	SBA	2006
	Measles	2006
	DPT1	2006
	DPT3	2006
	Fever	2006
	Diarrhea	2006
	ARI	–
Ethiopia		
	ANC	2000, 2005, 2011, 2016, 2019
	SBA	2011, 2016, 2019
	Measles	2000, 2005, 2011, 2016, 2019
	DPT1	2000, 2005, 2011, 2016, 2019
	DPT3	2000, 2005, 2011, 2016, 2019
	Fever	2000, 2005, 2011, 2016
	Diarrhea	2000, 2005, 2011, 2016
	ARI	2000, 2005, 2011, 2016
Gabon		
	ANC	2000, 2012
	SBA	2000, 2012
	Measles	2000, 2012
	DPT1	2000, 2012
	DPT3	2000, 2012
	Fever	2000, 2012
	Diarrhea	2000, 2012
	ARI	2000
Gambia		
	ANC	2013, 2019
	SBA	2013, 2019
	Measles	2013, 2019
	DPT1	2013, 2019
	DPT3	2013, 2019
	Fever	2013, 2019

	Diarrhea	2013, 2019
	ARI	2013, 2019
Ghana		
	ANC	1998, 2003, 2008, 2014, 2019
	SBA	1998, 2003, 2008, 2014
	Measles	1993, 1998, 2003, 2008, 2014
	DPT1	1993, 1998, 2003, 2008, 2014
	DPT3	1993, 1998, 2003, 2008, 2014
	Fever	1998, 2003, 2008, 2014, 2016, 2019
	Diarrhea	1998, 2003, 2008, 2014
	ARI	1998, 2003
Guinea		
	ANC	1999, 2005, 2012, 2018
	SBA	1999, 2005, 2012, 2018
	Measles	1999, 2005, 2012, 2018
	DPT1	1999, 2005, 2012, 2018
	DPT3	1999, 2005, 2012, 2018
	Fever	1999, 2005, 2012, 2018
	Diarrhea	1999, 2005, 2012, 2018
	ARI	1999, 2005, 2012
Kenya		
	ANC	1993, 2003, 2008, 2014, 2015
	SBA	1993, 2003, 2008, 2014
	Measles	1993, 1998, 2003, 2008, 2014
	DPT1	1993, 1998, 2003, 2008, 2014
	DPT3	1993, 1998, 2003, 2008, 2014
	Fever	1993, 2003, 2008, 2014, 2015
	Diarrhea	1993, 2003, 2008, 2014
	ARI	1993, 2003, 2008, 2014
Lesotho		
	ANC	2004, 2009, 2014
	SBA	2004, 2009, 2014
	Measles	2004, 2009, 2014
	DPT1	2004, 2009, 2014
	DPT3	2004, 2009, 2014
	Fever	2004, 2009, 2014
	Diarrhea	2004, 2009, 2014
	ARI	2004
Liberia		
	ANC	–

	SBA	2007, 2013, 2019
	Measles	2007, 2013, 2016, 2019
	DPT1	2007, 2013, 2016, 2019
	DPT3	2007, 2013, 2016, 2019
	Fever	2007, 2009, 2011, 2013, 2016, 2019
	Diarrhea	2007, 2013, 2019
	ARI	2007
Madagascar		
	ANC	–
	SBA	2004, 2008
	Measles	1997, 2004, 2008
	DPT1	1997, 2004, 2008
	DPT3	1997, 2004, 2008
	Fever	2004, 2008, 2011, 2013, 2016
	Diarrhea	2004, 2008
	ARI	2004, 2008
Malawi		
	ANC	–
	SBA	1992, 2000, 2004, 2010, 2015
	Measles	1992, 2000, 2004, 2010, 2015
	DPT1	1992, 2000, 2004, 2010, 2015
	DPT3	1992, 2000, 2004, 2010, 2015
	Fever	1992, 2000, 2004, 2010, 2012, 2014, 2015, 2017
	Diarrhea	1992, 2000, 2004, 2010, 2015
	ARI	1992, 2000, 2004, 2010, 2015
Mali		
	ANC	–
	SBA	2001, 2006, 2012, 2018
	Measles	1996, 2001, 2006, 2012, 2018
	DPT1	1996, 2001, 2006, 2012, 2018
	DPT3	1996, 2001, 2006, 2012, 2018
	Fever	2001, 2006, 2012, 2015, 2018
	Diarrhea	2001, 2006, 2012, 2018
	ARI	2001
Mauritania		
	ANC	–
	SBA	2000
	Measles	2000
	DPT1	2000
	DPT3	2000

	Fever	2000
	Diarrhea	2000
	ARI	2000
Mozambique		
	ANC	–
	SBA	2003, 2011
	Measles	1997, 2003, 2011, 2015
	DPT1	1997, 2003, 2011, 2015
	DPT3	1997, 2003, 2011, 2015
	Fever	2003, 2011, 2015, 2018
	Diarrhea	2003, 2011, 2015
	ARI	2003, 2015
Namibia		
	ANC	1992, 2000, 2006, 2013
	SBA	1992, 2000, 2006, 2013
	Measles	1992, 2000, 2006, 2013
	DPT1	1992, 2000, 2006, 2013
	DPT3	1992, 2000, 2006, 2013
	Fever	1992, 2000, 2006, 2013
	Diarrhea	1992, 2000, 2006, 2013
	ARI	1992, 2000
Niger		
	ANC	2006, 2012
	SBA	2006, 2012
	Measles	2006, 2012
	DPT1	1998, 2006, 2012
	DPT3	1998, 2006, 2012
	Fever	2006, 2012
	Diarrhea	2006, 2012
	ARI	2012
Nigeria		
	ANC	1990, 2003, 2008, 2013, 2018
	SBA	1990, 2003, 2008, 2013, 2018
	Measles	1990, 2003, 2008, 2013, 2018
	DPT1	1990, 2003, 2008, 2013, 2018
	DPT3	1990, 2003, 2008, 2013, 2018
	Fever	1990, 2003, 2008, 2010, 2013, 2015, 2018
	Diarrhea	1990, 2003, 2008, 2013, 2018
	ARI	1990, 2003, 2008, 2013, 2018

Rwanda		
	ANC	1992, 2000, 2005, 2008, 2010, 2015, 2019
	SBA	1992, 2000, 2005, 2008, 2010, 2015
	Measles	1992, 2000, 2005, 2008, 2010, 2015
	DPT1	1992, 2000, 2005, 2008, 2010, 2015
	DPT3	1992, 2000, 2005, 2008, 2010, 2015
	Fever	1992, 2000, 2005, 2008, 2010, 2013, 2015, 2017
	Diarrhea	1992, 2000, 2005, 2008, 2010, 2015
	ARI	1992, 2000, 2005, 2008, 2010, 2015
Sao Tome and Principe		
	ANC	2008
	SBA	2008
	Measles	2008
	DPT1	2008
	DPT3	2008
	Fever	2008
	Diarrhea	2008
	ARI	–
Senegal		
	ANC	1997, 2005, 2010, 2012, 2014-2019
	SBA	1997, 2005, 2010, 2012, 2014-2019
	Measles	2005, 2010, 2012, 2014-2019
	DPT1	2005, 2010, 2012, 2014-2019
	DPT3	2005, 2010, 2012, 2014-2019
	Fever	2005, 2006, 2008, 2010, 2012, 2014-2019
	Diarrhea	1997, 2005, 2010, 2012, 2014-2019
	ARI	2005, 2010, 2017
Sierra Leone		
	ANC	2008, 2013
	SBA	2008, 2013, 2019
	Measles	2008, 2013, 2019
	DPT1	2008, 2013, 2019
	DPT3	2008, 2013, 2019
	Fever	2008, 2013, 2016, 2019
	Diarrhea	2008, 2013, 2019
	ARI	2008, 2013
South Africa		
	ANC	1998, 2016
	SBA	1998, 2016
	Measles	1998, 2016

	DPT1	1998, 2016
	DPT3	1998, 2016
	Fever	2016
	Diarrhea	1998
	ARI	1998
Sudan		
	ANC	–
	SBA	1990
	Measles	1990
	DPT1	1990
	DPT3	1990
	Fever	–
	Diarrhea	–
	ARI	–
Tanzania		
	ANC	1996, 1999, 2004, 2010, 2015, 2017
	SBA	1996, 1999, 2004, 2010, 2012, 2015
	Measles	1996, 1999, 2004, 2010, 2015
	DPT1	1996, 1999, 2004, 2010, 2015
	DPT3	1996, 1999, 2004, 2010, 2015
	Fever	1996, 2004, 2007, 2010, 2012, 2015, 2017
	Diarrhea	1996, 2004, 2010, 2015
	ARI	1996, 2004, 2010, 2015
Togo		
	ANC	2013
	SBA	2013
	Measles	1998, 2013
	DPT1	1998, 2013
	DPT3	1998, 2013
	Fever	2013, 2017
	Diarrhea	2013
	ARI	–
Uganda		
	ANC	2000, 2006, 2011, 2016, 2018
	SBA	2000, 2006, 2011, 2016
	Measles	1995, 2000, 2006, 2011, 2016
	DPT1	1995, 2000, 2006, 2011, 2016
	DPT3	1995, 2000, 2006, 2011, 2016
	Fever	2000, 2006, 2009, 2011, 2014, 2016, 2018
	Diarrhea	2000, 2006, 2011, 2016

	ARI	2000, 2006, 2011, 2016
Zambia		
	ANC	1996, 2002, 2007, 2013, 2018
	SBA	1996, 2002, 2007, 2013, 2018
	Measles	1996, 2002, 2007, 2013, 2018
	DPT1	1996, 2002, 2007, 2013, 2018
	DPT3	1996, 2002, 2007, 2013, 2018
	Fever	1996, 2002, 2007, 2013, 2018
	Diarrhea	1996, 2002, 2007, 2013, 2018
	ARI	1996, 2002, 2007, 2013
Zimbabwe		
	ANC	1999, 2005, 2010, 2015
	SBA	1999, 2005, 2010, 2015
	Measles	1994, 1999, 2005, 2010, 2015
	DPT1	1994, 1999, 2005, 2010, 2015
	DPT3	1994, 1999, 2005, 2010, 2015
	Fever	1999, 2005, 2010, 2015
	Diarrhea	1999, 2005, 2010, 2015
	ARI	1999

DPT1 First-dose Diphtheria, Tetanus, Pertussis; *DPT3* Third-dose Diphtheria, Tetanus, Pertussis
ANC Antenatal Care; *ARI* Acute Respiratory Infection; *SBA* Skilled Birth Attendance

Table A2. Summary measures of coverage by 5-year intervals.

Indicator	Years	Mean (%)	Median (%)	Range (minimum, maximum) (%)	Range (quartile 1, quartile 3) (%)	SD (%)
ANC						
	1986-1990	52.4	51.9	(26.6, 85.4)	(36.0, 62.0)	19.0
	1991-1995	44.7	47.7	(8.0, 86.9)	(24.9, 62.6)	21.9
	1996-2000	45.1	48.9	(3.4, 87.6)	(22.8, 65.4)	23.9
	2001-2005	48.0	51.6	(1.5, 93.6)	(30.0, 66.4)	22.8
	2006-2010	51.0	49.3	(8.60, 94.7)	(36.0, 64.6)	18.9
	2011-2015	55.8	54.1	(6.8, 98.1)	(44.0, 71.8)	18.2
	2016-2019	59.8	58.7	(17.9, 98.9)	(47.8, 75.4)	18.3
SBA						
	1986-1990	42.1	40.1	(11.5, 78.9)	(25.0, 57.5)	20.5
	1991-1995	47.8	46.1	(5.0, 96.0)	(28.4, 64.0)	22.3
	1996-2000	49.8	46.9	(1.9, 98.7)	(27.0, 73.1)	26.6
	2001-2005	46.3	42.5	(1.0, 99.3)	(27.5, 66.5)	25.5
	2006-2010	58.6	61.0	(5.0, 98.2)	(39.9, 78.5)	24.0
	2011-2015	64.4	66.5	(2.1, 99.3)	(48.0, 87.6)	25.5
	2016-2019	72.9	79.6	(11.0, 100)	(60.0, 89.3)	22.1
Measles						
	1986-1990	43.56	42.7	(8.7, 72.0)	(33.3, 54.1)	18.3
	1991-1995	67.84	69	(23.4, 93.2)	(53.3, 84.1)	18.4
	1996-2000	62.76	65.05	(12.0, 95.2)	(46.0, 80.9)	21.2
	2001-2005	66.24	71.1	(8.2, 96.4)	(54.3, 83.2)	20.2
	2006-2010	77.49	82.45	(17.3, 97.4)	(67.0, 90.2)	16.4
	2011-2015	76.35	78.15	(13.2, 99.3)	(70.0, 87.1)	14.0
	2016-2019	74.82	78.5	(27.3, 97.4)	(65.7, 87.4)	16.3
DPT1						
	1986-1990	55.48	60.9	(9.9, 86.6)	(44.6, 69.5)	20.2
	1991-1995	82.28	87.5	(32.2, 99.0)	(75.9, 94.5)	15.7
	1996-2000	78.89	83.8	(18.8, 99.6)	(69.8, 94.3)	18.7
	2001-2005	80.95	87.75	(15.9, 99.7)	(72.4, 93.9)	18.1
	2006-2010	87.86	93.75	(22.6, 100.0)	(84.2, 96.4)	13.8
	2011-2015	87.56	92.15	(14.2, 100.0)	(83.8, 96.8)	13.6
	2016-2019	88.21	93.3	(40.2, 100.0)	(83.2, 96.9)	12.8

DPT3						
	1986-1990	36.37	30.5	(3.3, 71.7)	(21.3, 56.2)	22.0
	1991-1995	64.48	67.9	(20.3, 93.8)	(46.8, 86.2)	21.8
	1996-2000	59.34	65.1	(8.5, 95.7)	(40.7, 80.9)	24.3
	2001-2005	65.18	72.15	(4.7, 96.8)	(54.1, 82.5)	22.7
	2006-2010	75.99	83.45	(8.2, 98.7)	(66.4, 90.7)	19.3
	2011-2015	74.1	79.4	(7.0, 100.0)	(65.8, 87.9)	18.9
	2016-2019	75.54	78.05	(24.7, 99.7)	(65.6, 92.9)	17.8
Fever						
	1986-1990	67.05	67.8	(51.5, 84.8)	(58.0, 72.4)	10.9
	1991-1995	51.84	55.55	(16.4, 79.3)	(40.1, 79.3)	16.7
	1996-2000	34.62	28.55	(3.5, 94.2)	(19.9, 44.5)	19.5
	2001-2005	55.97	57.1	(10.9, 94.7)	(42.9, 69.9)	17.5
	2006-2010	64.41	64.5	(33.0, 93.7)	(55.5, 75.1)	13.5
	2011-2015	63.23	64.5	(17.3, 92.9)	(54.4, 73.4)	13.9
	2016-2019	65.9	68.5	(23.8, 95.2)	(56.2, 76.0)	13.7
Diarrhea						
	1986-1990	44.25	42.25	(32.2, 59.2)	(37.2, 48.6)	8.8
	1991-1995	44.18	40.25	(17.2, 77.4)	(31.8, 58.1)	16.2
	1996-2000	43.58	44.65	(15.5, 72.8)	(33.0, 53.5)	13.3
	2001-2005	40.81	40.95	(15.5, 65.7)	(32.4, 50.2)	12.1
	2006-2010	54.56	57.45	(18.2, 83.2)	(40.7, 65.8)	15.2
	2011-2015	57.45	56.7	(23.7, 82.6)	(49.3, 67.4)	11.5
	2016-2019	57.2	57.6	(28.2, 81.4)	(46.8, 66.9)	12.3
ARI						
	1986-1990	66.6	66.35	(53.5, 81.1)	(63.1, 69.1)	8.2
	1991-1995	58.27	62.2	(19.6, 92.9)	(41.3, 72.7)	18.3
	1996-2000	56.2	56.3	(11.5, 95.2)	(42.6, 73.1)	19.7
	2001-2005	60.28	63.2	(12.6, 94.2)	(48.8, 73.0)	18.0
	2006-2010	66.71	68.15	(23.4, 94.1)	(56.7, 77.6)	14.5
	2011-2015	64.43	67.1	(16.7, 97.0)	(54.6, 75.2)	15.4
	2016-2019	65.16	68.1	(23.6, 96.9)	(54.4, 78.2)	16.6

DPT1 First-dose Diphtheria, Tetanus, Pertussis; *DPT3* Third-dose Diphtheria, Tetanus, Pertussis
ANC Antenatal Care; *ARI* Acute Respiratory Infection; *SBA* Skilled Birth Attendance

Table A3. Linear model output by indicator and quintile.

Indicator	Quintile	Coefficient	Std. Error	P-value	R-squared
ANC					
	I	0.007	0.002	0.001	0.077
	II	0.007	0.002	0.000	0.084
	III	0.007	0.002	0.000	0.086
	IV	0.006	0.002	0.002	0.065
	V	0.004	0.002	0.029	0.032
SBA					
	I	0.012	0.002	0.000	0.173
	II	0.013	0.002	0.000	0.213
	III	0.014	0.002	0.000	0.222
	IV	0.011	0.002	0.000	0.161
	V	0.005	0.001	0.000	0.117
Measles					
	I	0.009	0.002	0.000	0.104
	II	0.007	0.002	0.000	0.089
	III	0.008	0.002	0.000	0.116
	IV	0.006	0.002	0.001	0.076
	V	0.003	0.001	0.036	0.033
DPT1					
	I	0.006	0.002	0.006	0.055
	II	0.006	0.002	0.002	0.069
	III	0.005	0.002	0.001	0.077
	IV	0.004	0.001	0.004	0.062
	V	0.001	0.001	0.098	0.020
DPT3					
	I	0.010	0.003	0.000	0.108
	II	0.010	0.002	0.000	0.105
	III	0.010	0.002	0.000	0.122
	IV	0.007	0.002	0.000	0.087
	V	0.003	0.001	0.052	0.028
Fever					
	I	0.011	0.002	0.000	0.212
	II	0.012	0.002	0.000	0.228

	III	0.012	0.002	0.000	0.227
	IV	0.011	0.002	0.000	0.215
	V	0.009	0.001	0.000	0.212
Diarrhea					
	I	0.009	0.001	0.000	0.206
	II	0.008	0.002	0.000	0.186
	III	0.009	0.002	0.000	0.184
	IV	0.007	0.001	0.000	0.129
	V	0.006	0.001	0.000	0.139
ARI					
	I	0.005	0.002	0.006	0.059
	II	0.005	0.002	0.005	0.061
	III	0.003	0.002	0.144	0.018
	IV	0.003	0.002	0.100	0.023
	V	0.003	0.002	0.055	0.034

DPT1 First-dose Diphtheria, Tetanus, Pertussis; *DPT3* Third-dose Diphtheria, Tetanus, Pertussis
ANC Antenatal Care; *ARI* Acute Respiratory Infection; *SBA* Skilled Birth Attendance

Figure A1. Trends by indicator for the six most populated countries in sub-Saharan Africa.

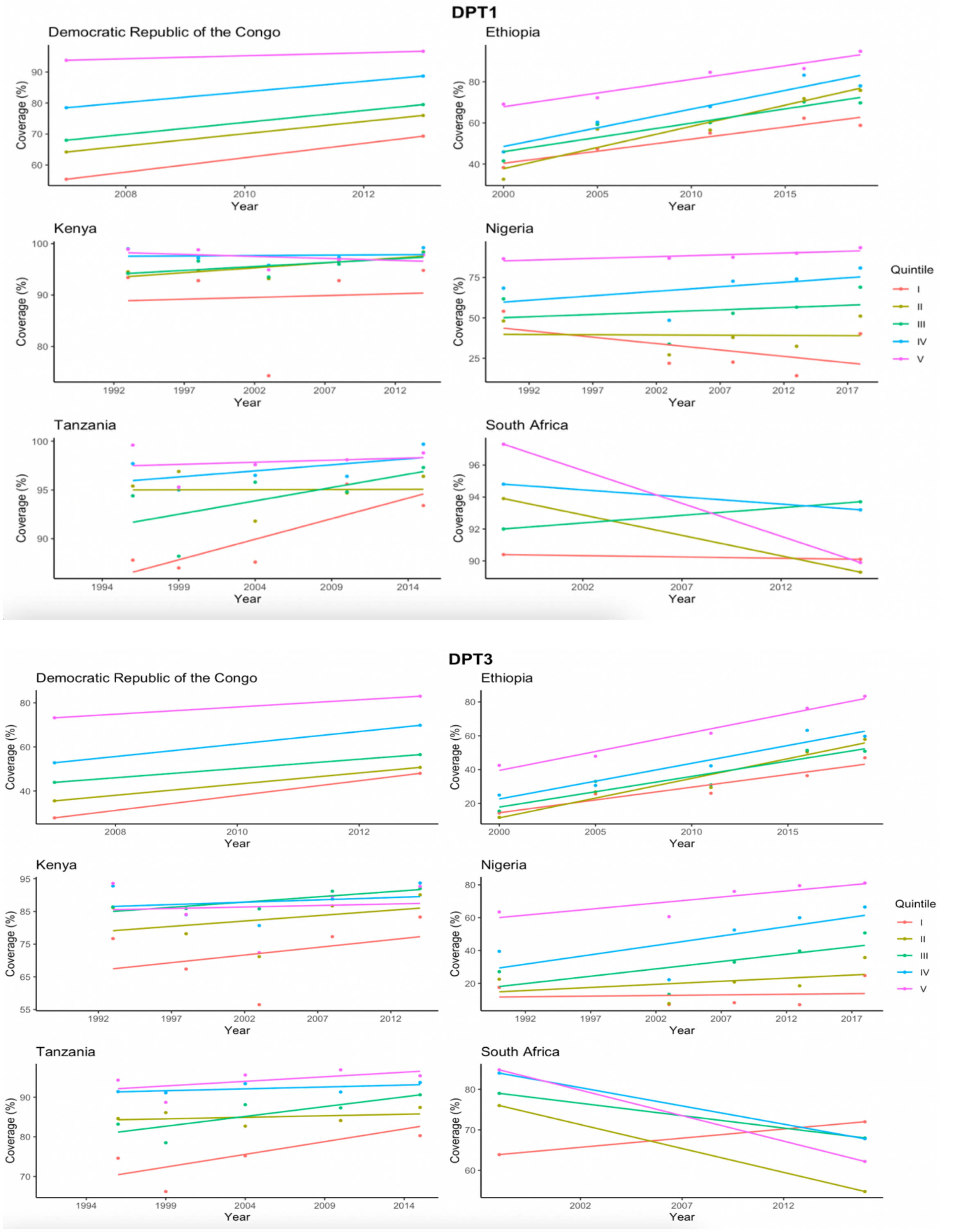

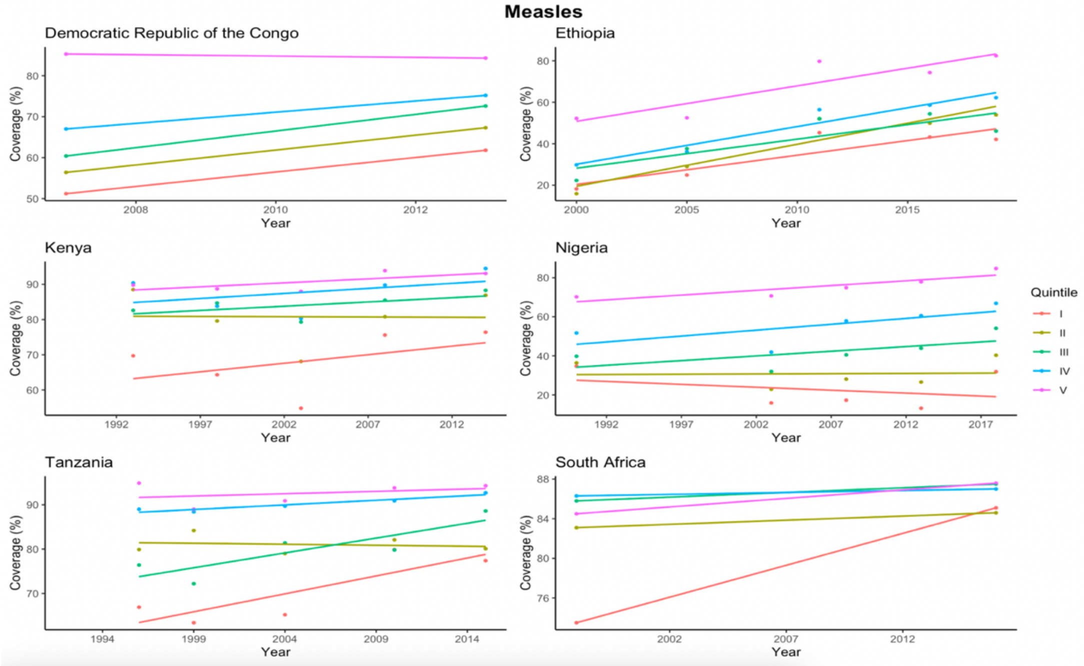
Measles
Democratic Republic of the Congo
Ethiopia
Kenya
Nigeria
Tanzania
South Africa
Coverage (%)
Year
Quintile
I
II
III
IV
V

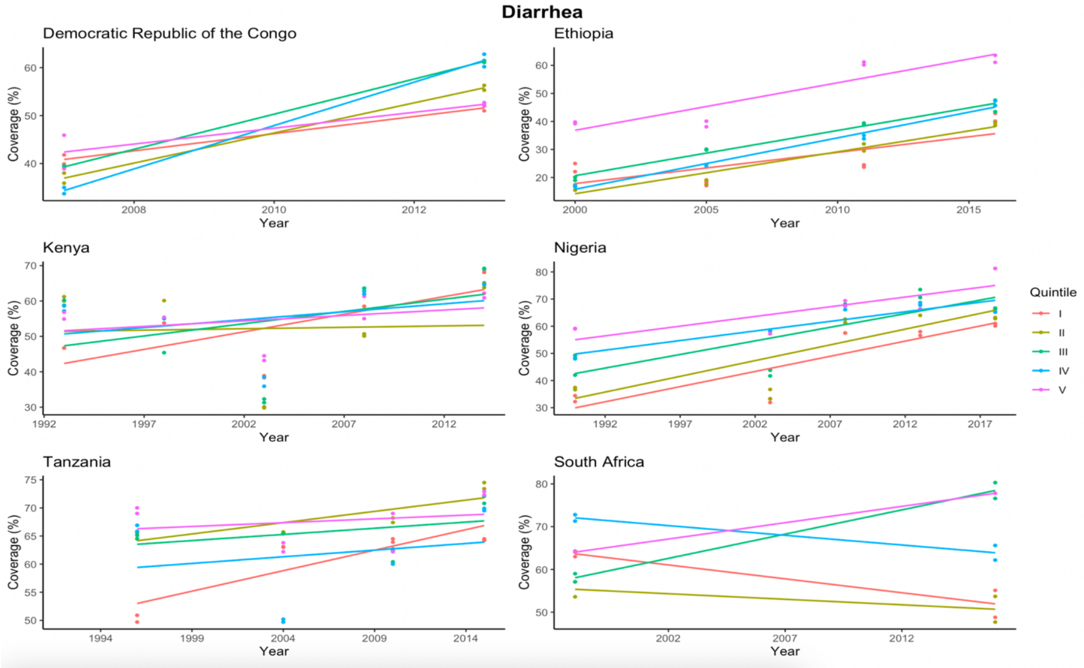
Diarrhea
Democratic Republic of the Congo
Ethiopia
Kenya
Nigeria
Tanzania
South Africa
Coverage (%)
Year
Quintile
I
II
III
IV
V

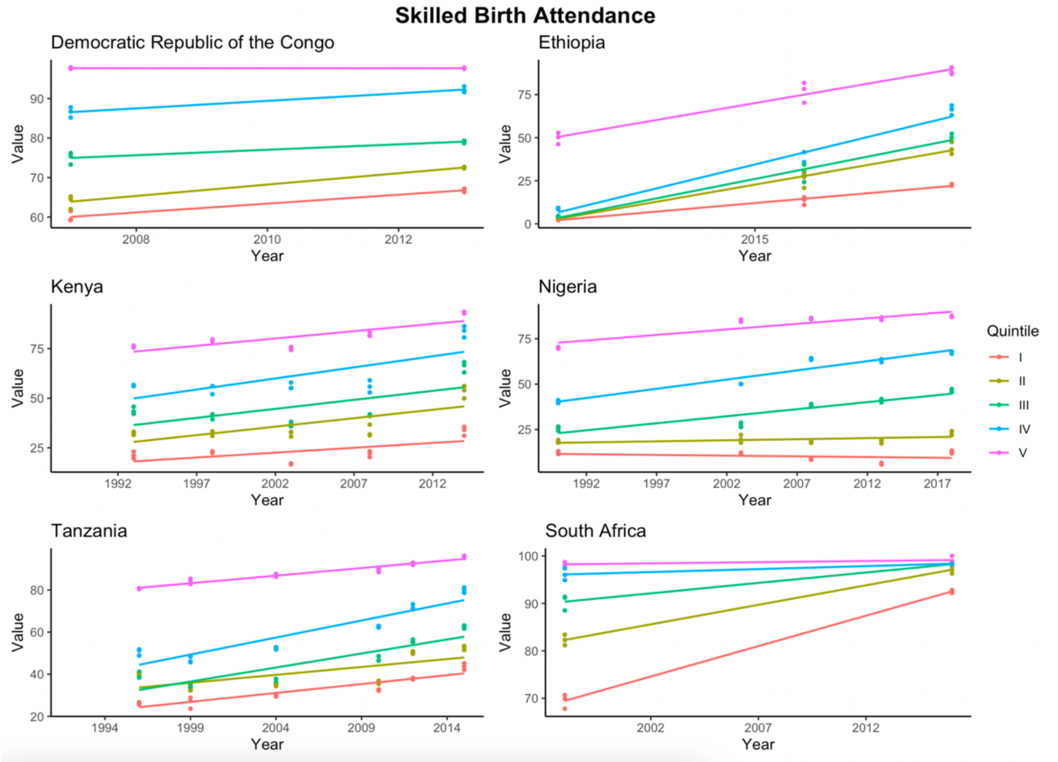
Skilled Birth Attendance
Democratic Republic of the Congo
Ethiopia
Kenya
Nigeria
Tanzania
South Africa
Value
Year
Quintile
I
II
III
IV
V

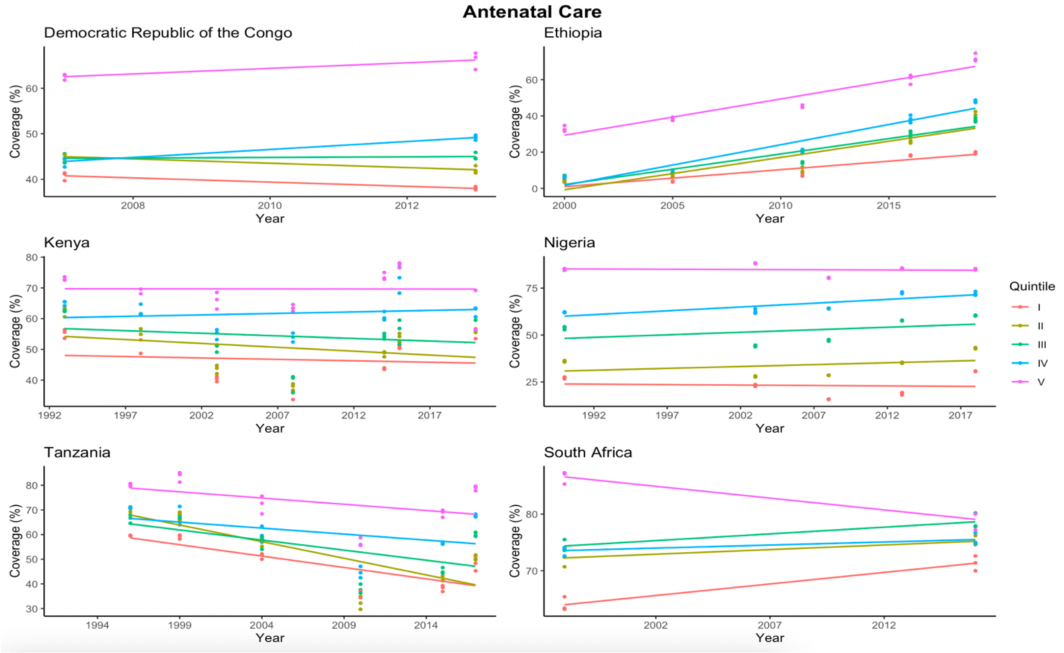
Antenatal Care
Democratic Republic of the Congo
Ethiopia
Kenya
Nigeria
Tanzania
South Africa
Coverage (%)
Year
Quintile
I
II
III
IV
V

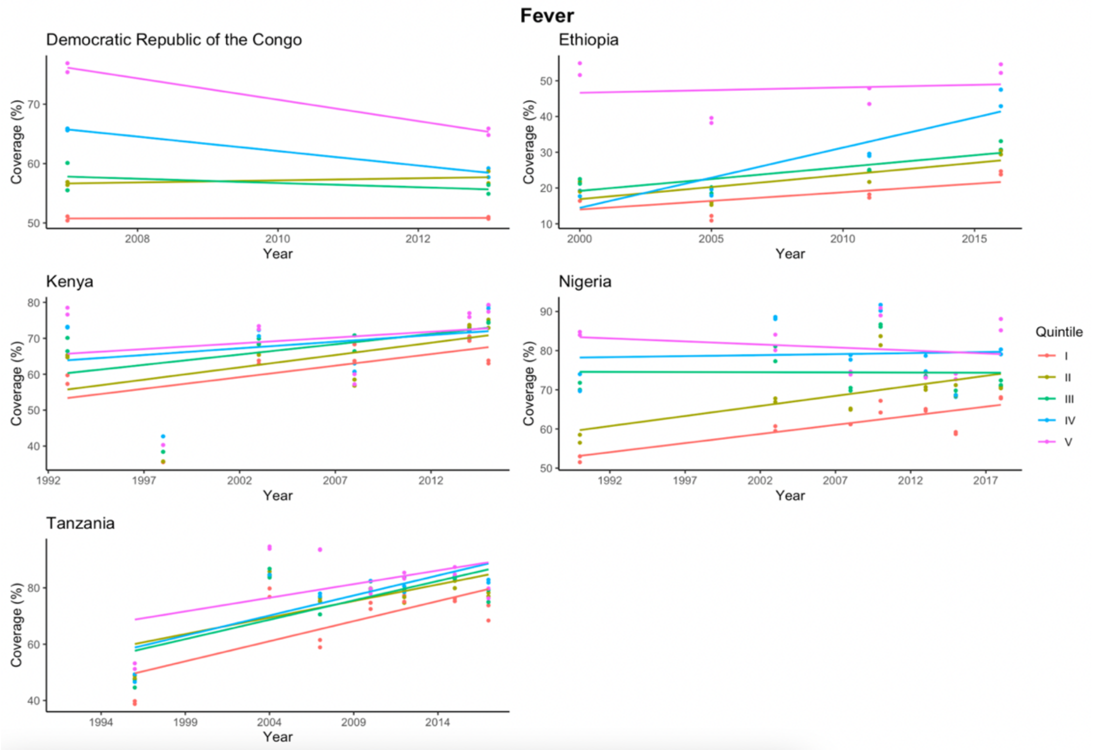
Fever
Democratic Republic of the Congo
Ethiopia
Kenya
Nigeria
Tanzania
Coverage (%)
Year
Quintile
I
II
III
IV
V

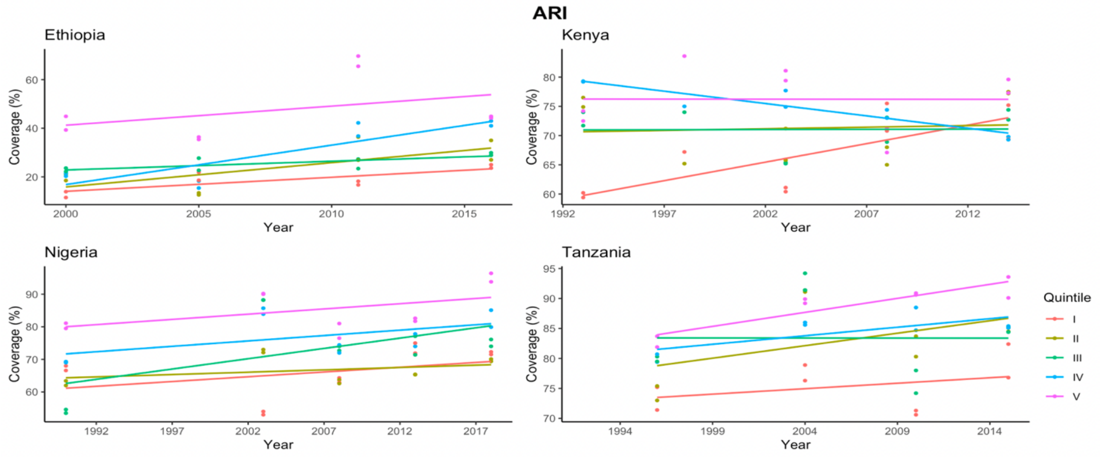
ARI
Ethiopia
Kenya
Nigeria
Tanzania
Coverage (%)
Year
Quintile
I
II
III
IV
V

Figure A2. Rate of change in coverage for the bottom quintile for eight indicators in sub-Saharan Africa from 1986 to 2019. All countries included present two or more surveys.

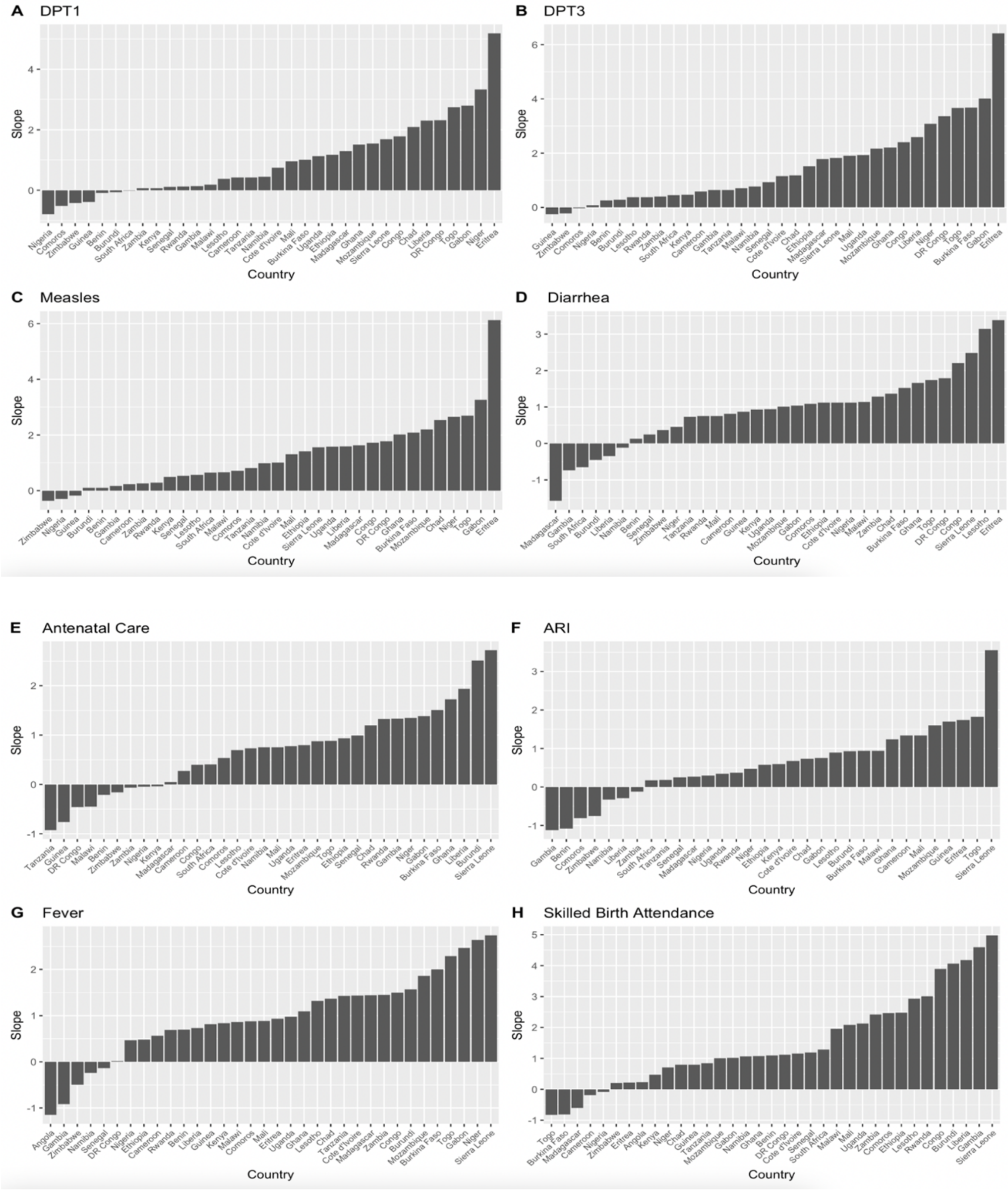

Slope: Average percentage point change between 1986 and 2019 for those in the bottom quintile from 1986 and 2019.

DPT1 First-dose Diphtheria, Tetanus, Pertussis; *DPT3* Third-dose Diphtheria, Tetanus, Pertussis
ANC Antenatal Care; *ARI* Acute Respiratory Infection; *SBA* Skilled Birth Attendance

Figure A3. Absolute differences and trends for eight indicators in sub-Saharan Africa, 1986-2019.

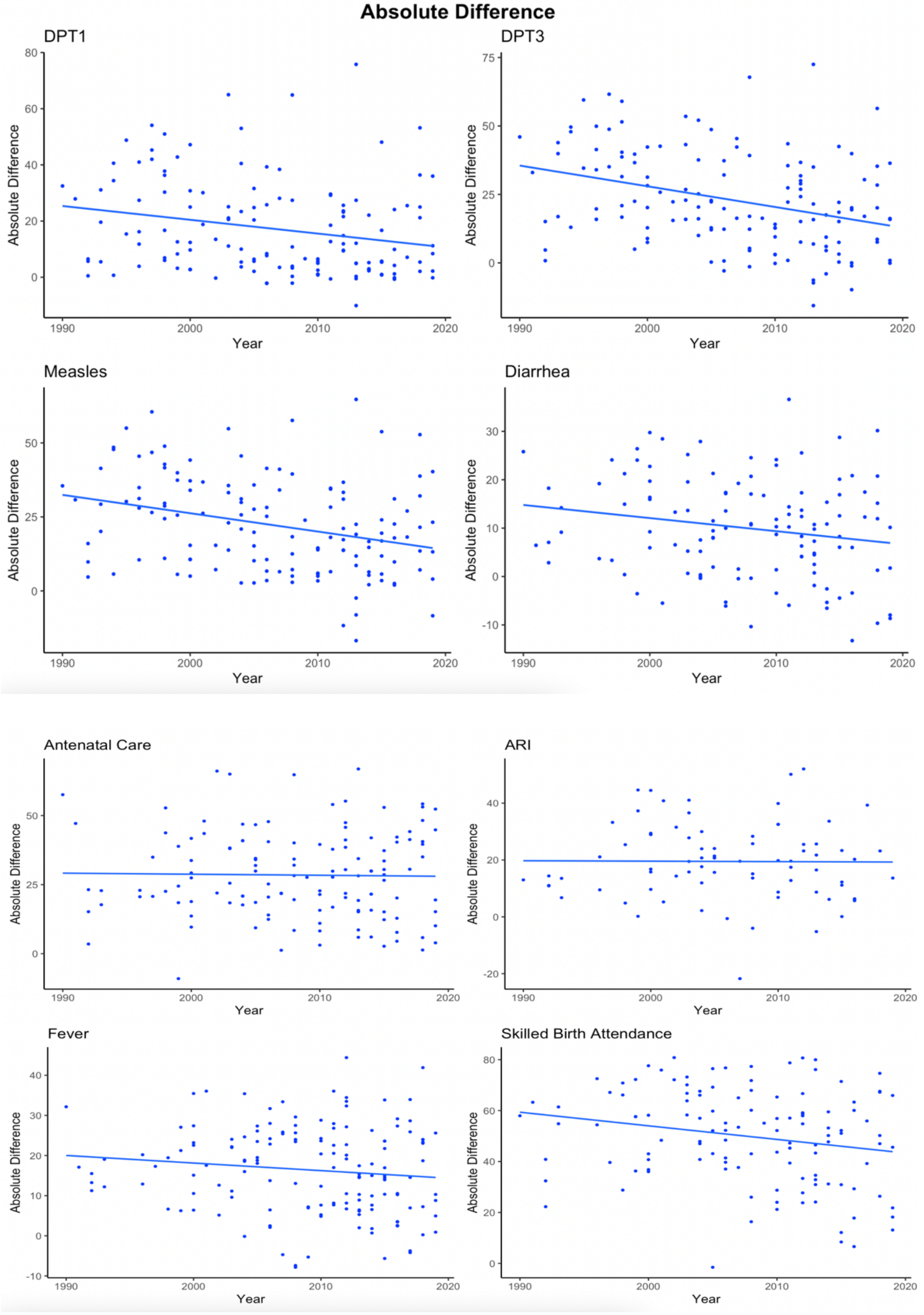

Note: The absolute difference is the difference between the top and bottom quintiles.

Figure A4. Relative differences and trends for eight indicators in sub-Saharan Africa, 1986-2019.

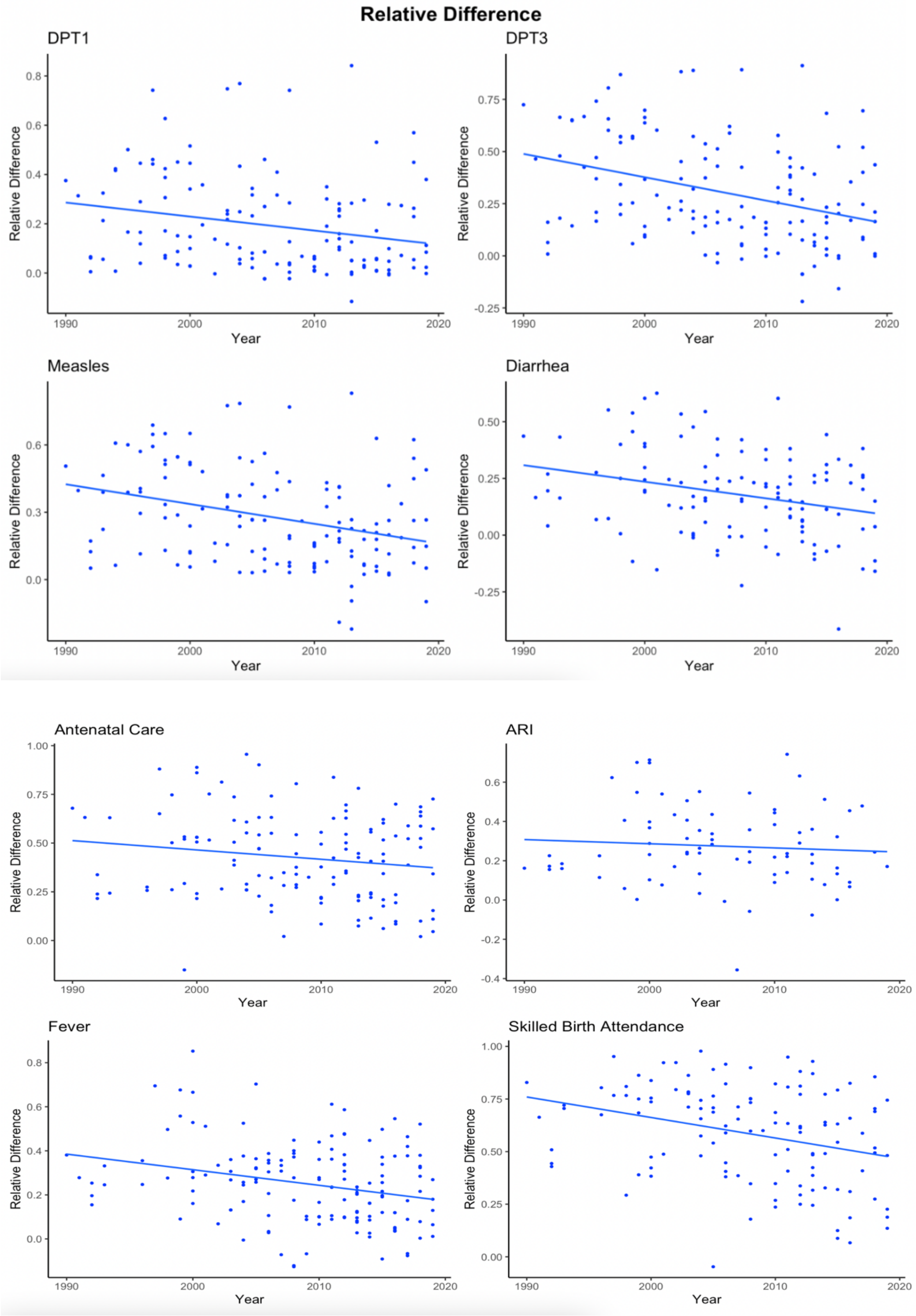

Note: The relative difference is the difference between the top and bottom quintiles divided by the top quintile.

Figure A5. Combined linear trends.

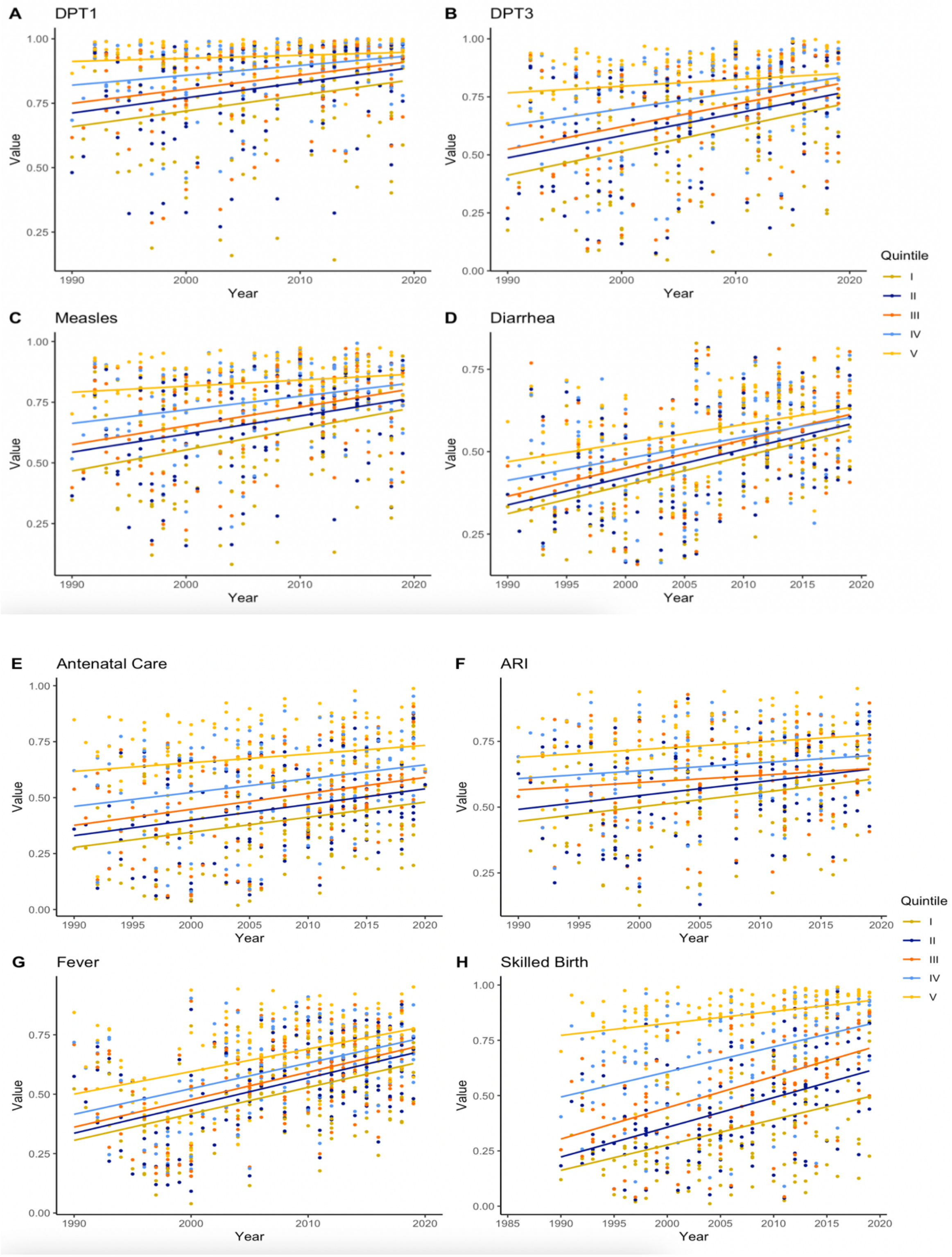

Figure A6. Density of the rate of change in coverage for the bottom quintile for eight indicators in sub-Saharan Africa, 1986 to 2019 for countries at least four surveys data points.

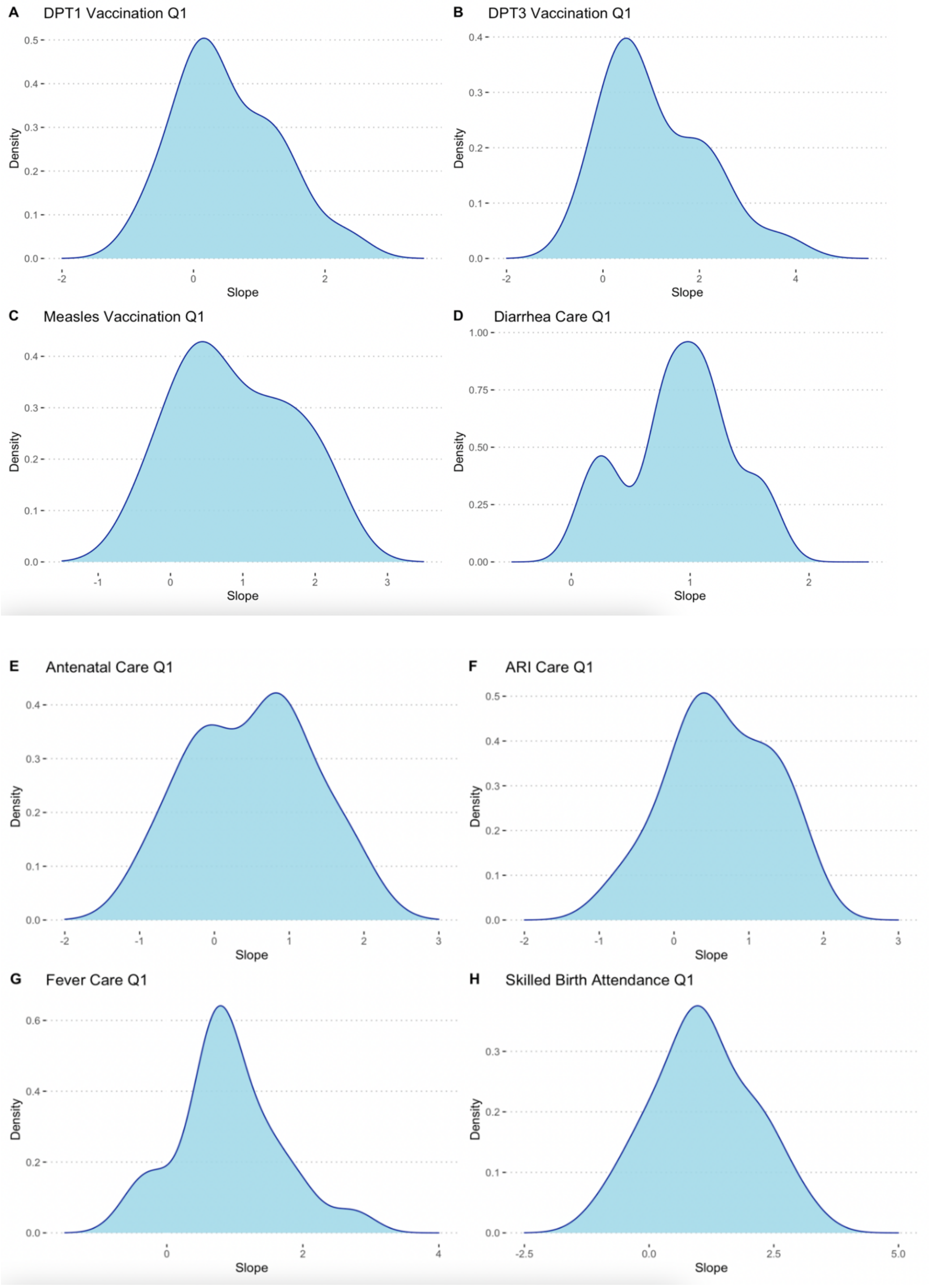

DPT1 First-dose Diphtheria, Tetanus, Pertussis; *DPT3* Third-dose Diphtheria, Tetanus, Pertussis
ANC Antenatal Care; *ARI* Acute Respiratory Infection; *SBA* Skilled Birth Attendance

Figure A7. Values of coefficient of variation and trends by quintile for eight indicators in sub-Saharan Africa, 1986-2019.

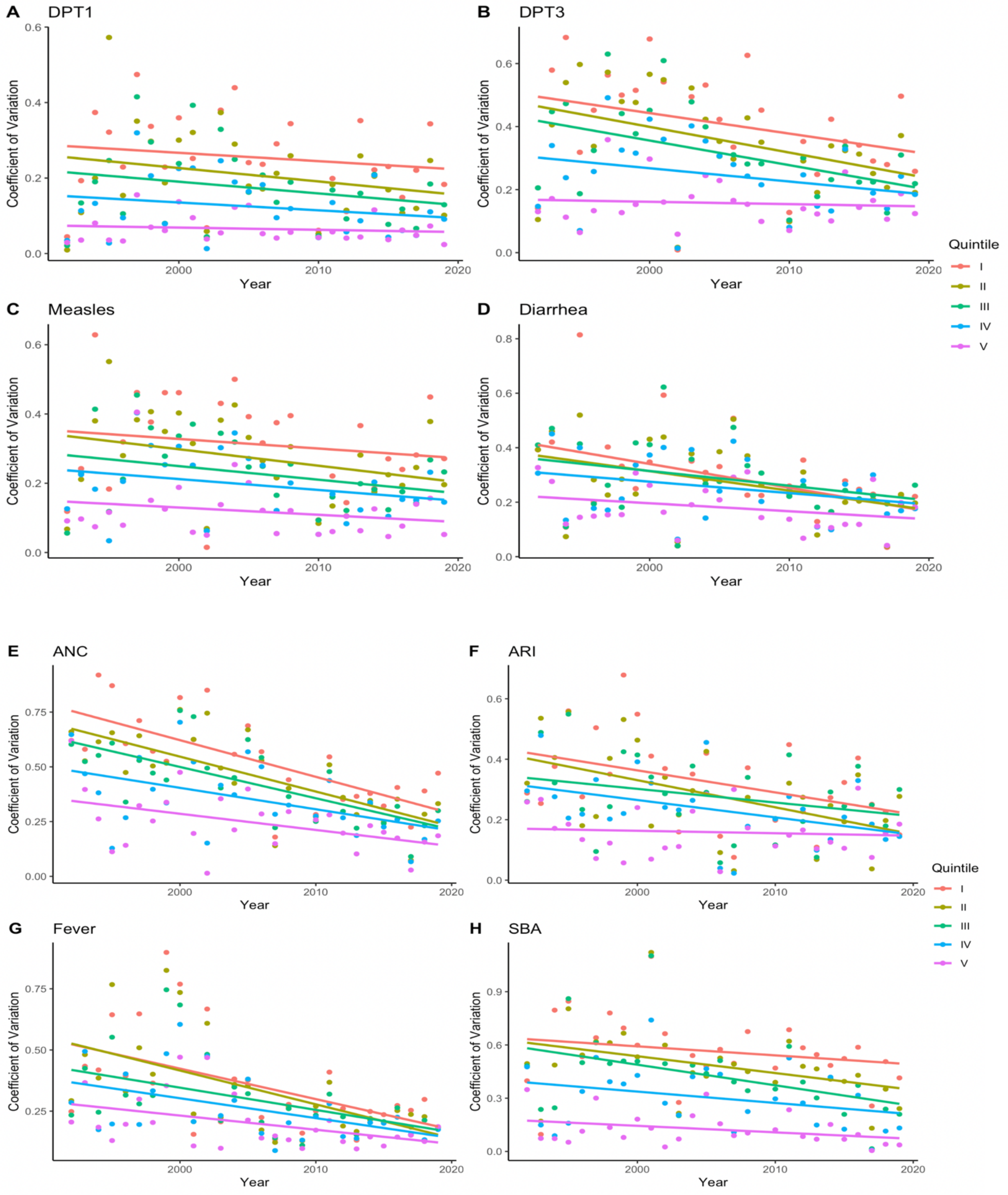

DPT1 First-dose Diphtheria, Tetanus, Pertussis; *DPT3* Third-dose Diphtheria, Tetanus, Pertussis
ANC Antenatal Care; *ARI* Acute Respiratory Infection; *SBA* Skilled Birth Attendance.

Figure A8. Five-year moving averages from 1986 to 2019 for eight indicators in sub-Saharan Africa with 25th and 75th percentile values.

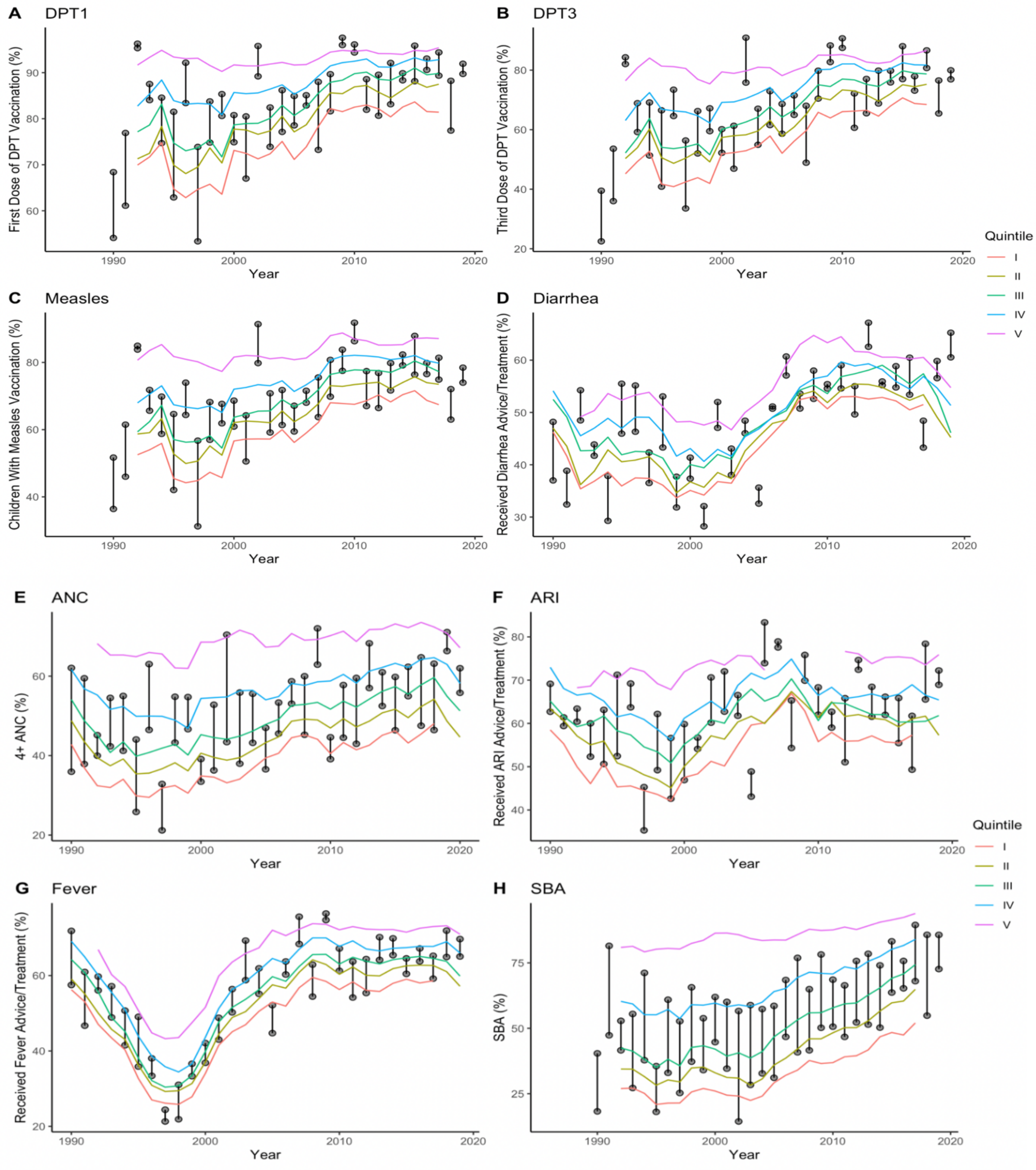

DPT1 First-dose Diphtheria, Tetanus, Pertussis; *DPT3* Third-dose Diphtheria, Tetanus, Pertussis
ANC Antenatal Care; *ARI* Acute Respiratory Infection; *SBA* Skilled Birth Attendance

Figure A9. Scatter plots and mean trends (1986-2019) for eight indicators in sub-Saharan Africa.

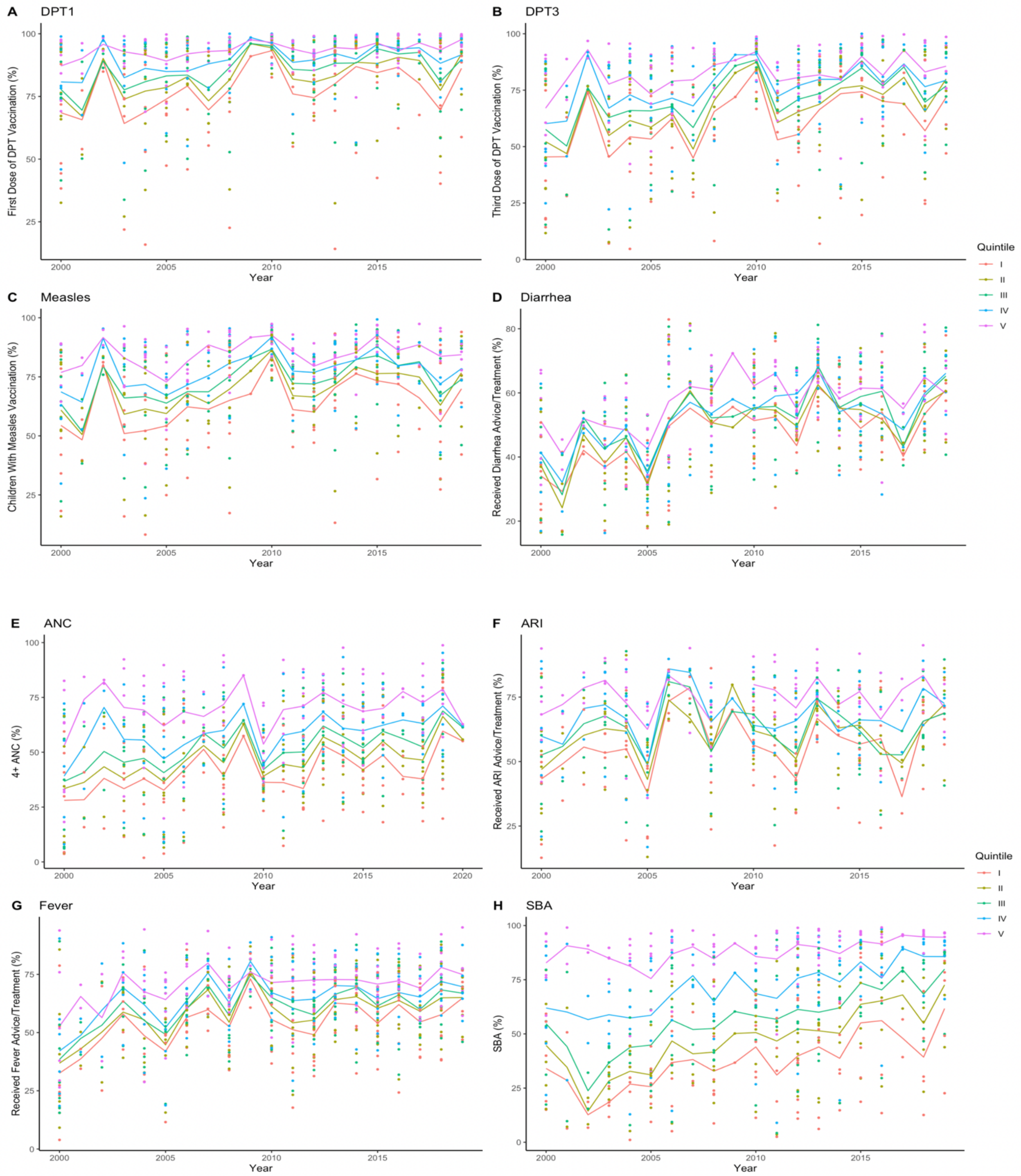

DPT1 First-dose Diphtheria, Tetanus, Pertussis; *DPT3* Third-dose Diphtheria, Tetanus, Pertussis
ANC Antenatal Care; *ARI* Acute Respiratory Infection; *SBA* Skilled Birth Attendance

Figure A10. Minimum and maximum values with five-year moving averages (1986-2019) for eight indicators in sub-Saharan Africa.

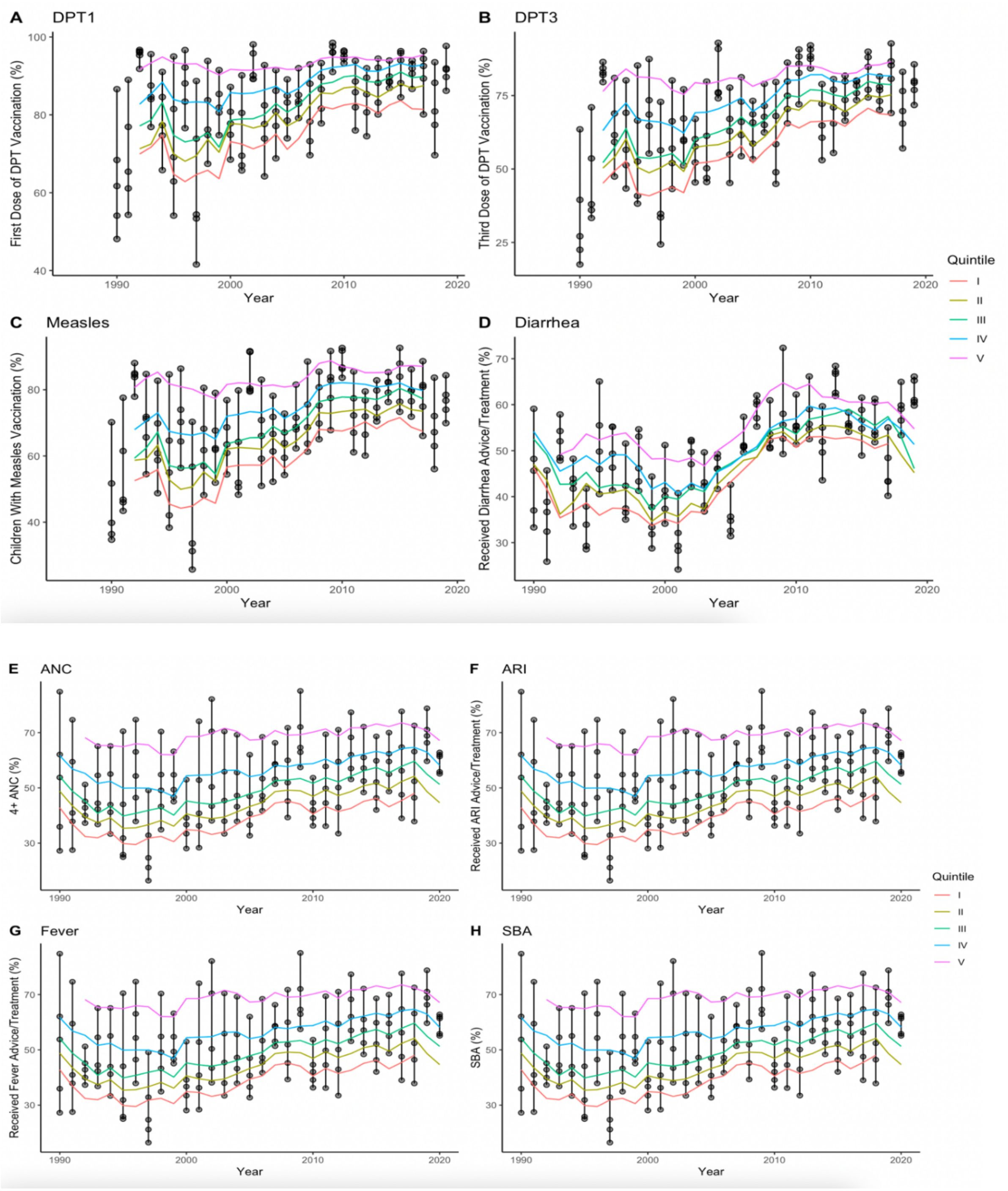

DPT1 First-dose Diphtheria, Tetanus, Pertussis; *DPT3* Third-dose Diphtheria, Tetanus, Pertussis
ANC Antenatal Care; *ARI* Acute Respiratory Infection; *SBA* Skilled Birth Attendance
